# Water-Soluble Sulfur-Ylide-Functionalized Polyacrylamides for Antibacterial Surface Applications

**DOI:** 10.1021/acs.langmuir.4c05134

**Published:** 2025-03-24

**Authors:** Bela B. Berking, Dimitrios Karagrigoriou, Daria R. Galimberti, Bai H. E. Zhang, Daniela A. Wilson, Kevin Neumann

**Affiliations:** † Systems Chemistry Department, Institute for Molecules and Materials, Radboud University, Heyendaalseweg 135, 6525 AJ Nijmegen, The Netherlands; ‡ Theoretical and Computational Chemistry Department, Institute for Molecules and Materials, Radboud University Nijmegen, Heyendaalseweg 135, 6525 AJ Nijmegen, The Netherlands

## Abstract

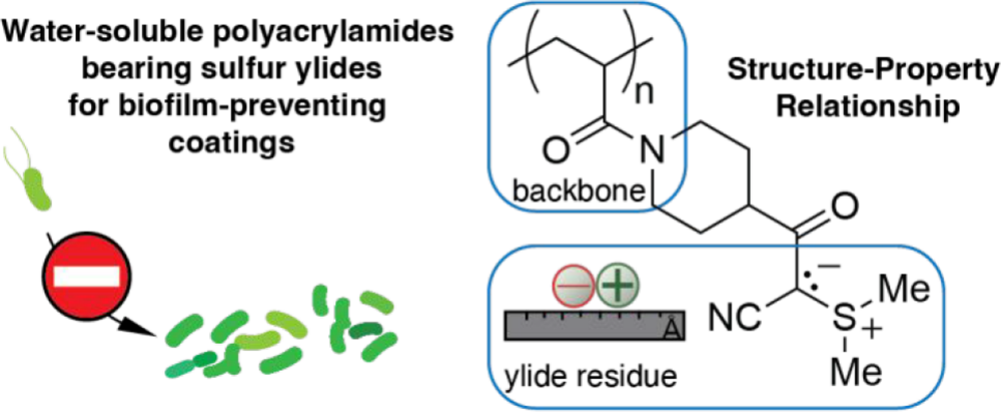

Surface fouling induced by biomolecules and microorganisms remains a persistent challenge in materials science, particularly in healthcare applications, where biofilm formation on medical devices may lead to infections and antimicrobial resistance. Antifouling strategies typically rely on the formation of either hydration layers or cytotoxic materials for direct antimicrobial effects. Recent advances in zwitterionic polymers derived from ylides offer a promising yet unexplored toolbox for the construction of antifouling and antimicrobial coatings. While N-oxide-based ylides have been extensively studied as building blocks for antifouling materials, sulfur-ylide-based materials, and the precise underlying mechanisms remain underexplored despite their broader chemical versatility. Here, we present a fully water-soluble acrylamide-based poly(sulfur ylide) and compare its properties to those of previously reported hydrophobic polystyrene-derived analogues. Notably, water-soluble poly(sulfur ylides) retain antimicrobial efficacy on surfaces but lose cytotoxicity in solution, unlike its hydrophobic counterpart. Computational studies reveal that the dipole moment of sulfur ylides is environmentally responsive, stabilizing in hydrophobic environments. Genetic analysis confirms outer membrane destabilization for both polymers but suggests that the hydrophobicity of the polystyrene backbone promotes stronger interactions. We suggest that future work should focus on elucidating additional interactions, including supramolecular behaviors of amphiphilic sulfur ylides, to refine their structure–property relationships and optimize their antifouling and antimicrobial properties.

## Introduction

Uncontrolled surface fouling by biomolecules and microorganisms remains a significant challenge in materials science, particularly for healthcare applications.^1,2^ In biological environments, biomolecules readily adhere to surfaces, acting as seeds for the subsequent attachment of microorganisms. This initial adhesion of single cells promotes the accumulation of further bacterial cells, ultimately leading to the formation of bacterial biofilms.^3^ These biofilms on medical devices, such as prosthetic joints, catheters, valves, and endotracheal tubes, can result in pathological infections and contribute to microbial resistance.^4,5^ To combat surface fouling and improve materials for healthcare, antifouling surfaces have been developed to prevent the nonspecific adhesion of biomolecules and microorganisms.^6,7^ Typically, antifouling surfaces are engineered either by forming hydration layers or by using cytotoxic components. Hydration layers act as physical barriers to prevent protein and microorganism attachment and are generally considered more biocompatible than cytotoxic coatings containing metals.^8^ This concept is also relevant in nanomedicine, where the nonspecific adsorption of blood proteins onto nanocarriers can enhance clearance and reduce drug delivery efficacy.^9,10^

**Figure 1:**
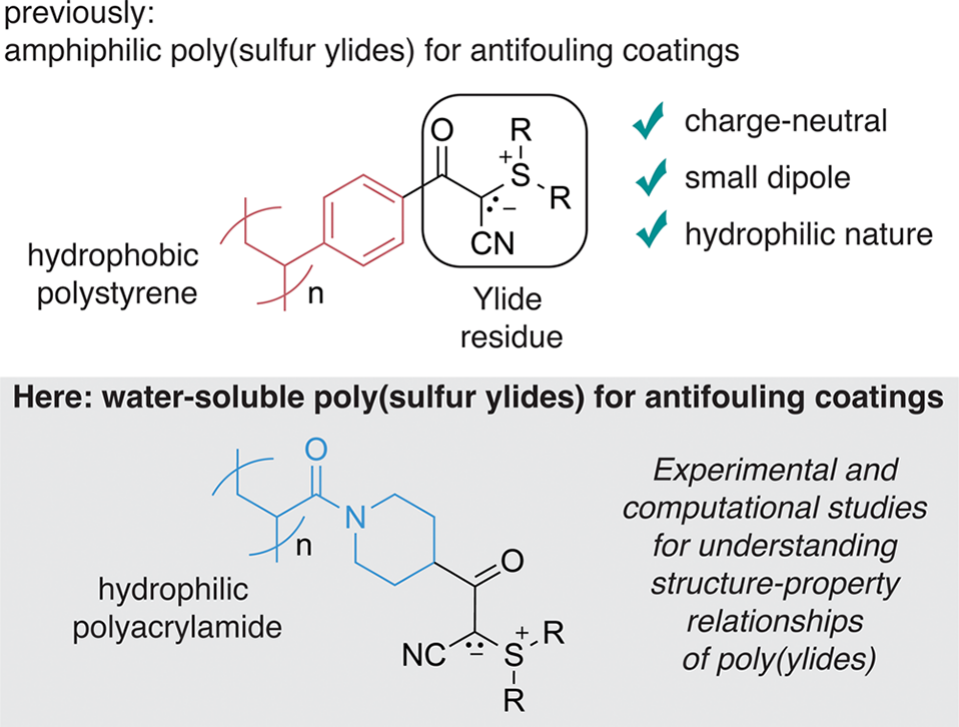
Poly(sulfur ylides) and poly(phosphorus ylides) have been reported as building blocks for antifouling coatings. Here, we report a fully water-soluble sulfur ylide displaying polyacrylamide, enabling the study of structure–property relationships and directing future research.

Installation of zwitterionic polymers, in particular, poly(betaines), onto surfaces is considered one of the most effective methods to establish hydration layers.^11,12^ This is because betaine-based zwitterions polymers solvate water electrostatically, thus forming strong hydration layers on the surface solution interface.^13^ Opposed to charged surfaces, which also display abilities to form strong hydration layers, zwitterionic coatings remain overall charge neutral, hence preventing electrostatic interactions with blood plasma proteins. The corresponding polymeric zwitterions can be grafted from the surface, for example, by using atom transfer radical polymerization. Alternatively, the polymers can be preformed and immobilized on the surface.^14,15^ The groups of Rosenhahn and Laschewsky elegantly used photo-crosslinking with a comonomer to enhance the stability and reproducibility of obtained surface coatings.^16,17^ Besides the choice of polymerization and surface attachment, the linker lengths that separate positive and negative influence the overall antifouling performance of zwitterionic coatings, with shorter linkers generally providing superior properties.^18^ In very insightful work, Wei and co-workers explained this observation with the smaller dipole displayed by zwitterionic scaffolds with shorter linkers, ultimately reducing dipole–dipole interactions with biomolecules.^19^ In pioneering work and inspired by the impact of the linker lengths, the group of Jiang reported dimethyl N-oxides bearing poly(acrylamides) as excellent building blocks for ultralow-fouling coatings.^20^ N-oxides possess a negatively charged oxygen directly adjacent to a positively charged ammonium, thus displaying the shortest available linker lengths.^21^ N-oxides coated surfaces displayed remarkable ultralow-fouling properties with no blood plasma proteins or other biomolecules adhered to the surface after exposure to blood serum as determined by surface plasma resonance. Computational studies revealed that oxygen atoms of trimethylammonium oxide accept on average 2.5 hydrogen bonds from water, thus building stronger interactions with water than polyethylene glycol. Recently, Maison and co-workers reported that N-oxides-bearing surfaces are not only efficiently forming hydration layers, but also display antimicrobial properties.^22^ The authors detected the formation of reactive oxygen species, which might explain the observed antimicrobial properties. Independently from the work on N-oxides, our groups reported the use of polymeric sulfur- and phosphorus ylides for the design of antimicrobial surfaces (Figure 1).^23−26^ In contrast to N-oxides, these ylides bear a negative charge on a carbon atom and offer a large but so far unexplored chemical space for the design of new materials. Interestingly, we observed bactericidal effects of both poly(styrene)-based sulfur and phosphorus ylides, while only little toxicity was observed toward mammalian cells. Due to their largely unexplored chemical space, these materials hold significant potential for future applications in materials science and drug delivery. However, the precise mechanisms underlying their bactericidal properties remain unclear, including the role of the polymer backbone, which has so far been limited to hydrophobic polystyrene.

Here, we investigate the structure–property relationship of these materials by accessing a water-soluble poly(acrylamide)-based form of poly(sulfur ylides). The goal is to detangle the roles of the formerly employed polystyrene backbone and ylide residues. While we determined a profound impact of ylide residues on the bacterial genome for both poly(acrylamide)- and poly(styrene)-based ylide materials, toxicity toward bacterial cells was only observed for on-surface immobilized ylides in the case of poly(SY-AAm), indicating a not completely innocent role of the poly(styrene) backbone.

With the help of computational studies, we propose a possible mechanism in which the ylide residue interacts with negatively charged cell membranes via dipole–dipole interactions and electrostatic binding of the sulfonium residues to membrane-displayed carboxylate residues. Notably, the dipole moment of ylide residues significantly decreases once exposed to hydrophobic environments, thus explaining the membrane-disruptive nature of those poly(ylides) with hydrophobic backbones.

## Experimental Section

### NMR Spectroscopy

Nuclear magnetic resonance (NMR) characterization was carried out on a Bruker AVANCE HD nanobay console with a 9.4 T Ascend magnet (400 MHz) and a Bruker AVANCE III console with a 11.7 T UltraShield Plus magnet (500 MHz) equipped with a Bruker Prodigy cryoprobe in chloroform (CDCl_3_) or DMSO-*d*_6_. NMR spectra were recorded at 298 K unless otherwise specified. Chemical shifts are given in parts per million (ppm) with respect to tetramethylsilane (TMS, δ 0.00 ppm) as an internal standard for ^1^H NMR. Coupling constants are reported as *J* values in Hz. Peak assignment is based on the 2D ^1^H–^1^H COSY, ^1^H–^13^C HSQC, and ^1^H–^13^C HMBC spectra. The splitting patterns are indicated as follows: s, singlet; br. s, broad singlet; d, doublet; t, triplet; m, multiplet.

### Contact Angle Measurements

For contact angle measurements, polymers were covalently attached on an amine-coated glass surface (ESI). The contact angle measurements were performed on an FTA 1000 drop shape instrument (B frame system) equipped with a Basler acA720-290gm camera and Navitar coupler with lens (1-60135). Three microliters of Milli-Q water were placed on the coated polymer surface and then snapshots were taken, and the contact angle was determined using *imagej*.

### Synthesis of Poly(SY-AAm)

In a typical experiment, a Schlenk finger was purged with argon and charged with a RAFT agent (1.0 equiv). Monomer **1** (49.0 equiv) was added under Ar and dissolved in anhydrous DMF (1M) under stirring. Then, AIBN (0.1 equiv) and trioxane (0.25 M) were added as initiator and internal standard, respectively. The solution was degassed for 20 min and a sample for NMR analysis was taken (*t* = 0 h). The solution was heated to 80 °C and the reaction was monitored by NMR. After the desired conversion was indicated (70%), the reaction solution was allowed to reach room temperature and exposed to air. The product was precipitated dropwise in cold Et_2_O, redissolved in methanol, and subsequently precipitated dropwise in cold Et_2_O. The product was dried under a high vacuum to afford the desired polymer as a yellow solid (83%).

### Bacterial Adhesion

The overnight culture was inoculated in 6 mL of brain heart infusion (BHI) by adding 5 μL of *P. aeruginosa* ATCC 10145, 50% Glycerol stock, and incubated overnight at 37 °C. The next day, the resulting culture was diluted to an OD of 0.01 and was seeded to allow for biofilms to grow. The plates were incubated at 37 °C.

### RNA Extraction

Bacteria from overnight cultures were diluted and incubated after various treatments. RNA was extracted by using a standard RNA extraction kit. Cells were lysed using a bead homogenizer, and the lysate was processed through spin columns with multiple washing and centrifugation steps to purify the RNA. The RNA was eluted with RNase-free water. The concentration and purity of the RNA were measured by using a spectrophotometer, and the presence of specific RNA was confirmed by agarose gel electrophoresis.

## Computational Section

**Figure 2:**
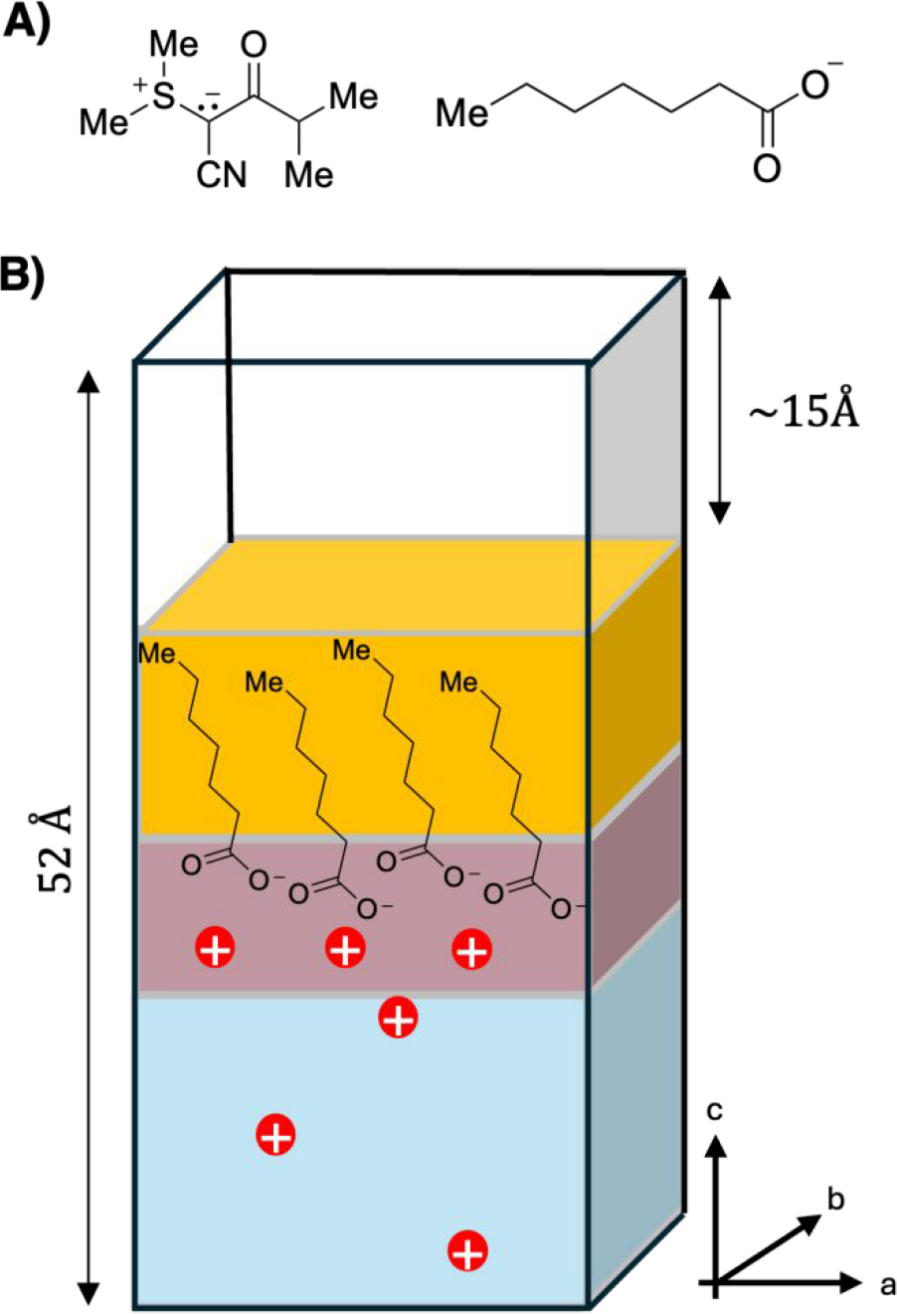
(A) Ylide residue and heptanoates used for the described calculation. (B) Scheme of the simulation box. The red dots represent potassium cations, the blue region represents the water solution, the white region is the vacuum space, the yellow region represents the hydrophobic lipid layer, and the violet region is the interfacial region, as identified by our free energy profile.

We designed a simplified model of a negatively charged water–lipid interface and simulated the penetration of a single ylide residue (Figure 2). The lipid layer has been modeled as a monolayer of nine heptanoic carboxylates. The aqueous solution in contact with the lipid layer consists of a water slab of 256 water molecules. Nine potassium cations were added to the liquid water phase as counterions. Periodic boundary conditions were applied in all three spatial directions to mimic a bulk liquid phase. Additionally, a vacuum space of ∼15 Å was added in the *c*-direction to separate the water phase from the tails of heptanoates.

The free energy profile was obtained by a metadynamics-enhanced sampling technique using Density Functional Tight Binding Born–Oppenheimer Molecular Dynamics (DFTB-MD) simulations.^27−33^ In particular, we selected the eXtended Tight Binding GFN1-xTB description.^34^ At each time step, the electronic wave function is converged. The forces acting on the classical nuclei are computed. The classical Newton equations of motion for the nuclei are integrated through the velocity Verlet algorithm with a time step of 0.5 fs. The choice of DFTB-MD simulations instead of the more accurate Density Functional Theory-MD (DFT-MD) simulation has been forced by the dimension of the system (999 atoms) and the required computational time to converge the free energy profile (around 1.5 ns). All the DFTB-MD have been run with the Cp2k code^35^ coupled with the PLUMED^36^ interface for the biased MD. The simulations have been run in the NPT_F ensemble, i.e., constant temperature and pressure, using a flexible simulation box. In particular, the simulation box was allowed to relax and change shape along the *x* and *y* directions, but we fixed it along the *z* to maintain the vertical vacuum space; i.e., the *a*, *b*, and γ cell parameters were free to change, while *c*, α, and β were kept fixed. For all the simulations, the average temperature of the system was set to 300 K using a CSVR thermostat^37^ with a time damping constant of 300 fs. The average pressure along *x* and *y* was set to 1 atm using a barostat with a time damping constant of 1 ps.

As a first step, we constructed a water–lipid interface. We started with a simulation box with dimensions of 15.6 Å × 15.6 Å × 52.0 Å, comprising the water solution and the heptanoates but not the ylide. We equilibrated the sample for 80 ps. As a second step, the ylide was inserted into the center of the water slab, and the system was equilibrated again. Starting from the equilibrated state, we have run an OPES metadynamics^38^ still in the NPT_F ensemble, i.e., the membrane can open up to accommodate the ylide molecule. We selected the distance along *z* between the center of mass of the COO^–^ heads of the heptanoates and the center of mass of the ylide molecules as reaction coordinates. The initial guess for the barrier to overcome was 100 kJ/mol. The frequency for kernel deposition was set to 200 steps. Two walls were placed at −7.5 Å (lipid layer side) and 14.5 Å (water solution side) to restrain exploration of the molecule around the interfacial region. The metadynamics was run for a total simulation time of around 1.5 ns. The free energy profile so obtained shows three distinct minima.

As a last step, the stability of these minima was confirmed by unbiased DFT-MD simulations. All of the DFT-MD was run again with the Cp2k code. The free energy profile so obtained shows three clear and distinct minima. As a last step, their stability was confirmed by a set of unbiased DFT-MD simulations. All the DFT-MD have been run again with the Cp2k code. We used the BLYP functional augmented with the D2 dispersion corrections.^39,40^ A hybrid Gaussian and plane waves (GPW) basis set, consisting of a 350 Ry energy cutoff plane-wave basis set, coupled with the DZVP-MOLOPT-SR-GTH basis set, was selected.^41^ Pseudopotentials of the GTH type (Goedecker-Teter-Hutter)^42^ were also adopted. Six initial states (atomic positions, velocities, and cell parameters) were extracted from the metadynamics simulations: two with the molecule inside the lipid layer and four with the molecule in the interfacial minimum on the waterside. As a compromise with the computational cost, the number of water molecules was reduced to 141, removing molecules from the vacuum-water interface side and the c-cell parameter was reduced accordingly. The volume was fixed, and the simulations were run in the NVT, i.e., constant temperature and volume ensemble. For all of the simulations, the average temperature of the system was set to 300 K using a CSVR thermostat with a time-damping constant of 300 fs. We equilibrated the simulations box for 5 ps and then ran at least 25 ps of the production.

For both the two simulations with the ylide inside the lipid layer as a starting point, the ylide either is stable within the lipid layer or tends to diffuse toward the vacuum. Of the four simulations starting at the interfacial minimum, in one, the ylide molecule moved toward the bulk of the water solution; in one, the ylide passed spontaneously inside the lipid layer; in two, the ylide was stable at the interface. We extended one of these letters for an additional 25 ps (for a total of 50 ps of simulation time) to confirm the presence of the minimum definitively. The ylide was shown to be stable at the interfacial minimum for more than 45 ps. Only in the last picosecond, it started to diffuse toward bulk water minimum.

## Results and Discussion

**Figure 3:**
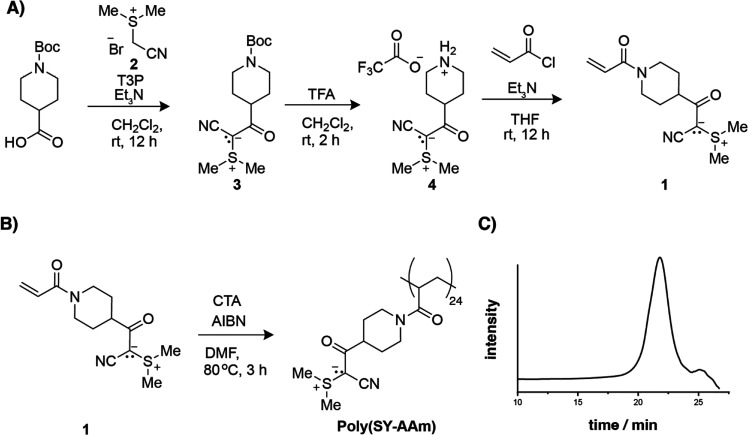
(A) Synthetic route toward sulfur ylide bearing acrylamide monomer **1**. (B) Polymerization was achieved using 2-[[(2-Carboxyethyl)sulfanylthiocarbonyl]-sulfanyl]propanoic acid as chain transfer agent providing Poly(SY-AAm) with an average of 24 repeating units. (C) Analysis with size exclusion chromatography revealed monomodal size distributions of poly(SY-AAm).

At the onset of our studies, we accessed polymeric sulfur ylides displaying an acrylamide backbone for elucidating the impact of the polymeric backbone on the efficiency of the reported poly(sulfur ylides) to serve as antimicrobial coatings. For this purpose, we designed acrylamide monomer **1** which we could access in three steps starting from commercially available *N*-Boc protected isonipecotic acid and sulfonium salt **2** (Figure 3). Initial ylide formation using T3P provided compound **3**. After subsequent Boc-removal using TFA/CH_2_Cl_2_, the obtained sulfur ylide **4** was coupled to acryloyl chloride to give sulfur ylide acrylamide **1**. This monomer was polymerized using reversible addition–fragmentation chain-transfer (RAFT) conditions with trithiocarbonate propanoic acid as a chain transfer agent. Due to the high polarity of the acrylamide monomer, polymerization was carried out in DMF at 80 °C. The resulting poly(sulfur ylide acrylamide) (poly(SY-AAm)) displayed high polarity and was soluble in pure water as well as in polar, protic solvents, confirming our hypothesis that an acrylamide backbone enhances overall water solubility.

**Figure 4:**
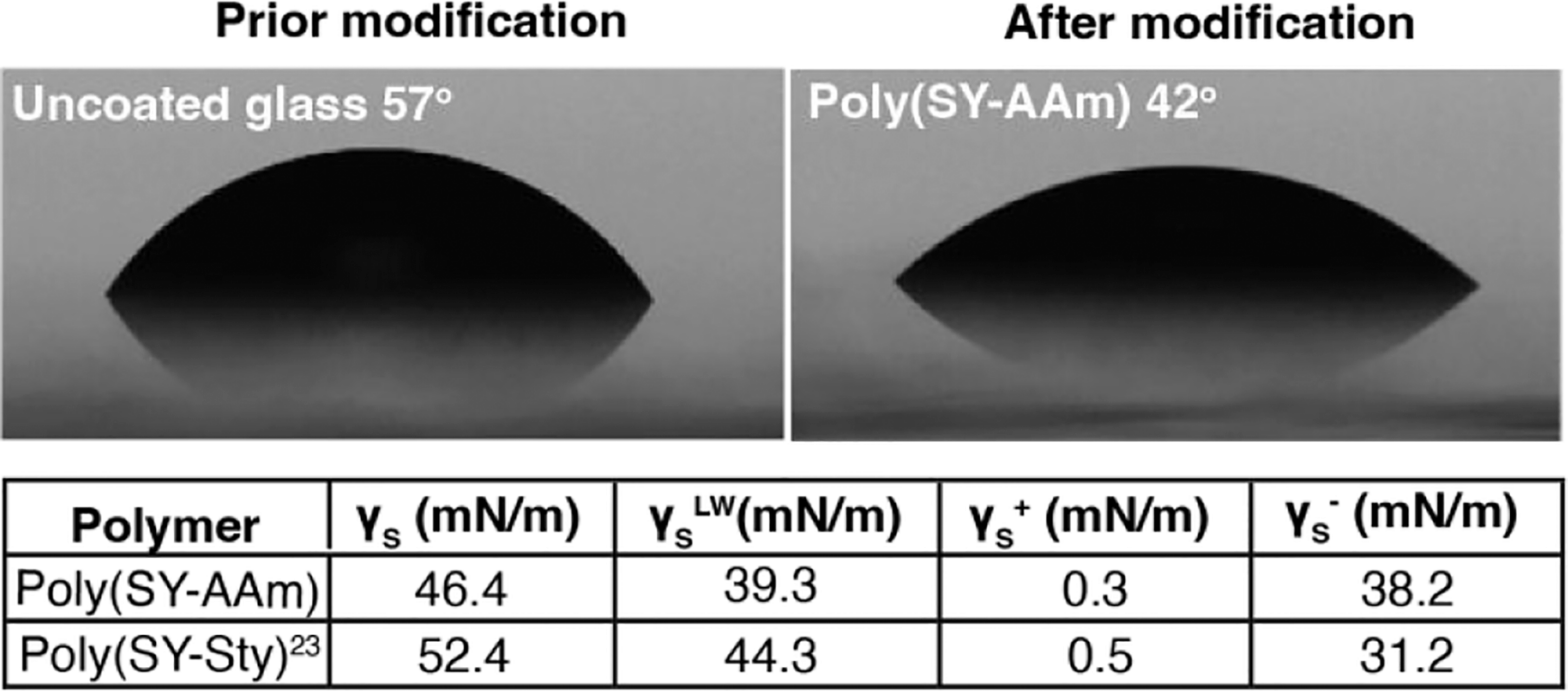
Poly(SY-AAm) modified glass surfaces displayed a decreased water contact angle. Compared to literature-reported polystyrene sulfur ylides poly(SY-Sty), surfaces displayed a smaller water contact angle and lower surface energy.

Hydration layers and resulting wettability are key requirements for the design of antifouling surfaces. Thus, we investigated the ability of poly(SY-AAm) to induce hydration layers by determining the wettability and conducting surface energy analyses. For this purpose, we covalently immobilized poly(SY-AAm) on amine-displaying glass surfaces using 1-ethyl-3-(3-(dimethylamino)propyl)carbodiimide chemistry. The obtained contact angles Θ_c_ = 42° indicated a more hydrophilic character of the surfaces than previously reported poly(styrene)-derived ylides residues (Θ_c_ = 52°), although wettability is less strong than for some superhydrophilic zwitterionic surfaces such as N-oxide derived coatings (Figure 4).^20,23^ To further elucidate the properties of poly(SY-AAm) displaying surfaces, we carried out a surface energy analysis. Somewhat surprisingly, poly(SY-AAm) modified surfaces displayed a lower surface energy γ_S_ of 46.4 mN/m than previously reported poly(styrene)-derived sulfur ylides (poly(SY-Sty)).^23^ We hypothesize that fully water-soluble poly(SY-AAm) are more ordered on the surfaces, likely supported by amide residues as well as the high water-solubility; in contrast to the poly(styrene) backbones which likely collapse on surfaces, potentially resulting in a more ordered surface and less surface energy. Notably, we observed a significantly high Lewis base component γ_S_^–^ = 38.2 mN/m indicating not only a strong ability to accept hydrogen bonds and possibly attributing to the overall ability to form hydration layers but also fulfilling the Whitesides rules.^43^

**Figure 5:**
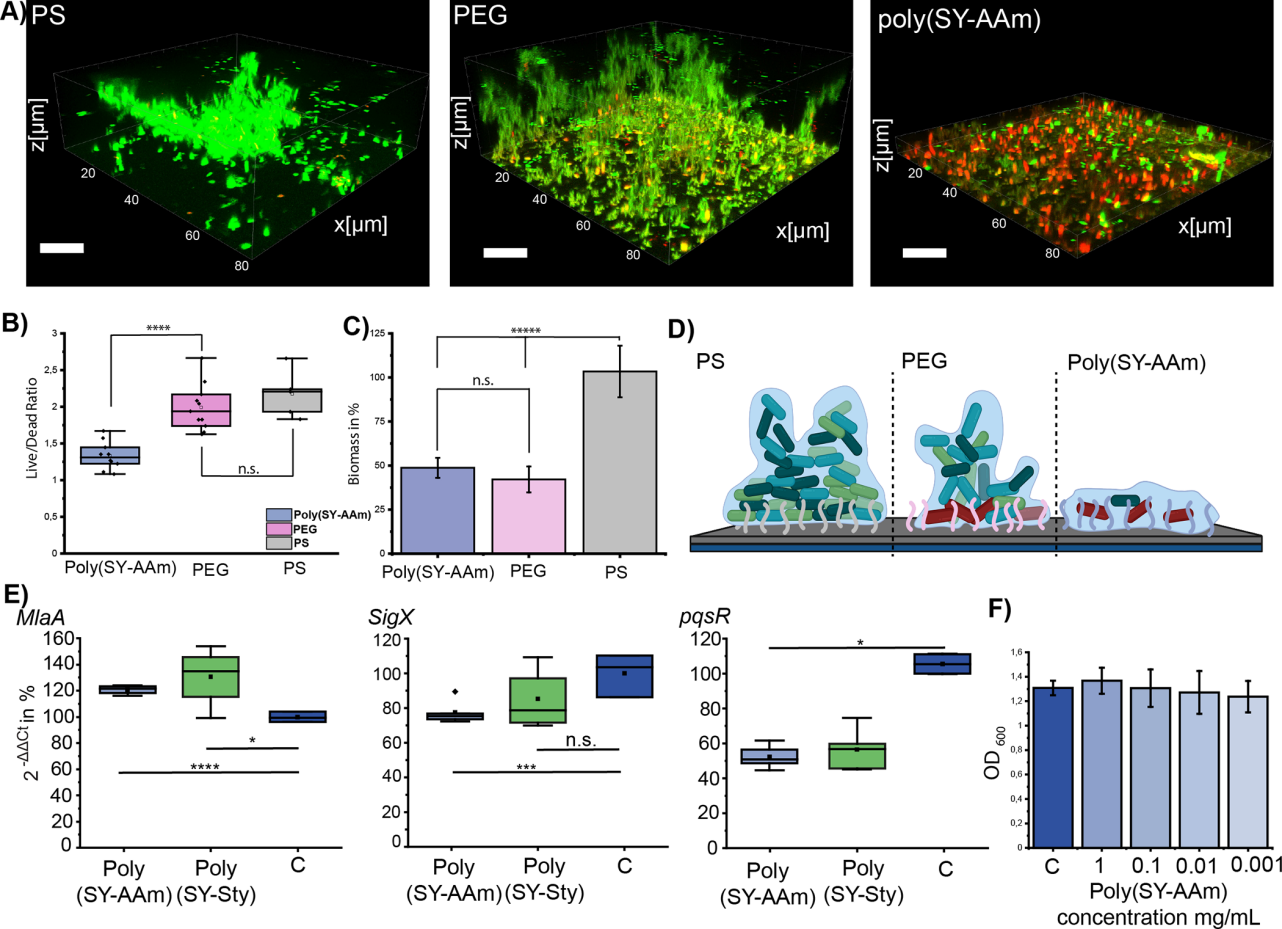
Effects of poly(SY-Sty) and poly(SY-AAm) coated surfaces on biofilm formation: (A) Confocal images of biofilms grown on polystyrene, PEG and poly(SY-AAm), scale bars are set at 20 μm. (B) Live/dead ratio of biofilms, quantified via Biofilm Viability Checker from confocal images. (C) Biomass measured using the Crystal Violet assay. Statistical significance was assessed via f.test and one-sided t.test with *p* ≤ 0.01. Schematic overview of different biofilm growth developments on the three tested surfaces. (D) Proposed mechanism of poly(SY-AAm). (E) qRT-PCR analysis of genes MlaA, sigX and pqsR. Statistical significance was assessed via f.test and one-sided t.test with *p* ≤ 0.05. Graphical overview of molecular response to poly(SY-AAm), upregulation of MlaA for outer membrane lipid transfer following outer membrane damage, and disruption of SigX and pqsR. (F) Viability assays of planktonic *P. aeruginosa* treated with poly(SY-AAm) with OD control: 1.31, 1 mg/mL: 1.37, 0.1 mg/mL: 1.31, 0.01 mg/mL: 1.27 and 0.001 mg/mL: 1.24. Parts of the figure were created with biorender.

To investigate the potential effects of poly(SY-AAm) on biofilm formation, amine-bearing glass surfaces were functionalized with PEG, PS, and poly(SY-AAm) using 1-Ethyl-3-(3-(dimethylamino)propyl)carbodiimide chemistries, followed by incubation with Gram-negative *P. aeruginosa* to promote biofilm growth. Confocal images revealed fully formed biofilms with complex pores on PS and PEG surfaces. In contrast, bacteria were unable to grow biofilms on poly(SY-AAm) surfaces beyond one or two cell layers, with the majority of cells being nonviable (Figure 5A). This effect was quantified by calculating the live/dead ratio (Figure 5B), which showed a significantly less viable bacterial population on poly(SY-AAm) compared to that on both PEG- and PS-modified surfaces. As expected, the overall biomass accumulation on poly(SY-AAm)-coated surfaces was significantly reduced (Figure 5C). Although confocal images showed functional biofilms on PEG-coated surfaces, quantification revealed a lower overall biomass compared to PS.

We hypothesize that while PS provides an ideal hydrophobic surface, PEG primarily inhibits initial attachment but has a minimal effect on the viability of cells that overcome the initial hydration layer. These surviving cells can then seed biomass accumulation, eventually leading to biofilm overgrowth (Figure 5D). For obtaining additional insights into bacterial responses, the underlying genetic responses activated by poly(SY-AAm) were compared to those of styrenic poly(SY-Sty). Since earlier reports suggest that poly(SY-Sty) is causing membrane damage,^23,25^ we analyzed the transcript levels of the outer membrane lipoprotein MlaA after 4 h of exposure to either poly(SY-AAm) or poly(SY-Sty). The Mla (maintenance of outer membrane lipid asymmetry) apparatus has been shown to aid in outer membrane lipid asymmetry, with MlaA effectively removing mislocalized glycerophospholipids from the outer membrane.^44,45^ Bacterial cells exposed to either poly(SY-AAm) or poly(SY-Sty), displayed significantly increased MlaA transcripts when compared to nontreated controls (Figure 5E). This finding indicates the disturbance of the outer membrane by both polymers. Many genetic responses and adaptations in *P. aeruginosa* are controlled by transcriptional regulators called sigma factors.^46^ Among these sigma factors, SigX is a main regulator when it comes to the response to cell wall envelope stress, as well as the production of virulence factors such as alginate.^47,48^ We observed that upon exposure to the ylide polymers, SigX transcription levels are significantly decreased for poly(SY-AAm) treatment yet do not significantly change for poly(SY-Sty). SigX has been shown to be upregulated upon exposure to most cell-wall-targeting compounds; therefore, the decrease in transcriptional activity seems quite counterintuitive. Some data suggest that decreasing SigX results in higher baseline biofilm formation, therefore one plausible hypothesis is a potential feedback loop, in which downregulation is meant to compensate for the lack of biofilm formation.^49^ Other work highlights the connection between the loss of SigX and membrane fluidity and integrity.^50^ Considering these observations, we believe that there is an underlying mechanistic difference in how poly(SY-Sty) and poly(SY-AAm) interact with the membrane due to the difference in chemical structure arising from the altered polymer backbone. Interestingly, no change in MlaA transcription was observed upon incubation with PEG, further highlighting the unique properties of poly(ylides) (Figure S7).

A big hurdle when combating biofilms is the secreted virulence factors, allowing for adaptation of the bacterial population to adverse effects, the host immune system, and other parameters dictating the infection cycle.^51^ The pseudomonas quinolone signal (pqs) system is a main contributor to the excretion of such virulence factors, as well as a core regulator of the quorum sensing network, which governs biofilm growth and dispersion.^52,53^ The adverse effects of both poly(SY-AAm) and poly(SY-Sty) significantly decrease the transcription levels of pqsR, which stands at the top of the pqs system, by controlling the production of the pqs signal molecule, as well as activating the excretion of toxins and other pathways.^54,55^ The inhibition of the pqs system is a highly desirable effect due to the direct consequence this has on biofilm virulence, persisted cells, and overall viability, all being negatively affected by shutting down this network.^56^

Our results highlight the capabilities of both water-soluble poly(SY-AAm) and the poorly water-soluble poly(SY-Sty) to prevent biofilm formation when applied as coatings, in particular the tendency to induce cell death to bacterial cells that adhere to coated surfaces. Interestingly, when applied in solution, we did not observe induced cell death of planktonic cells treated with water-soluble poly(SY-AAm) (Figure 5F). While analysis of genetic responses revealed some similarities in the impact of both ylide-bearing polymers on Gram-negative *P. aeruginosa*, we could also identify differences, for example, the downregulation of SigX. While these results suggest a distinct behavior of ylide functionalities at the interface of water and cell membrane, our results indicate also a noninnocent role of the styrene backbone.

To better understand the specific role of the ylide residue in the interaction between poly(SY-AAm) and negatively charged membranes and to ultimately correlate the observed properties with its molecular structure, we developed a simplified water–lipid interface model for computational analysis. The purpose of this model was to provide insights into the ylide behaviors at hydrophobic/hydrophilic interfaces. The interface was modeled as a monolayer of heptanoates in contact with bulk water (Figure 2B). Heptanoic acid was chosen as a relatively small lipid that sought to provide sufficient hydrophobic interactions to form monolayers with the capability of embedding small molecules. Thus, it can be considered as a compromise between accuracy and reasonable computational costs. Potassium was added to the water liquid phase as a counterion. While the simplicity of this model and the selected level of theory do not allow quantitative analysis, they provide a qualitative view of interactions at a negatively charged interface between aqueous and hydrophobic environments. Upon simulation, we observed the formation of an electric double layer at the interface.

Potassium cations accumulate on the surface and form a first layer that is directly adsorbed on and within the polar and negatively charged RCOO^–^ groups, followed by a diffuse layer to screen the negatively charged surface of the model lipid layer.

**Figure 6:**
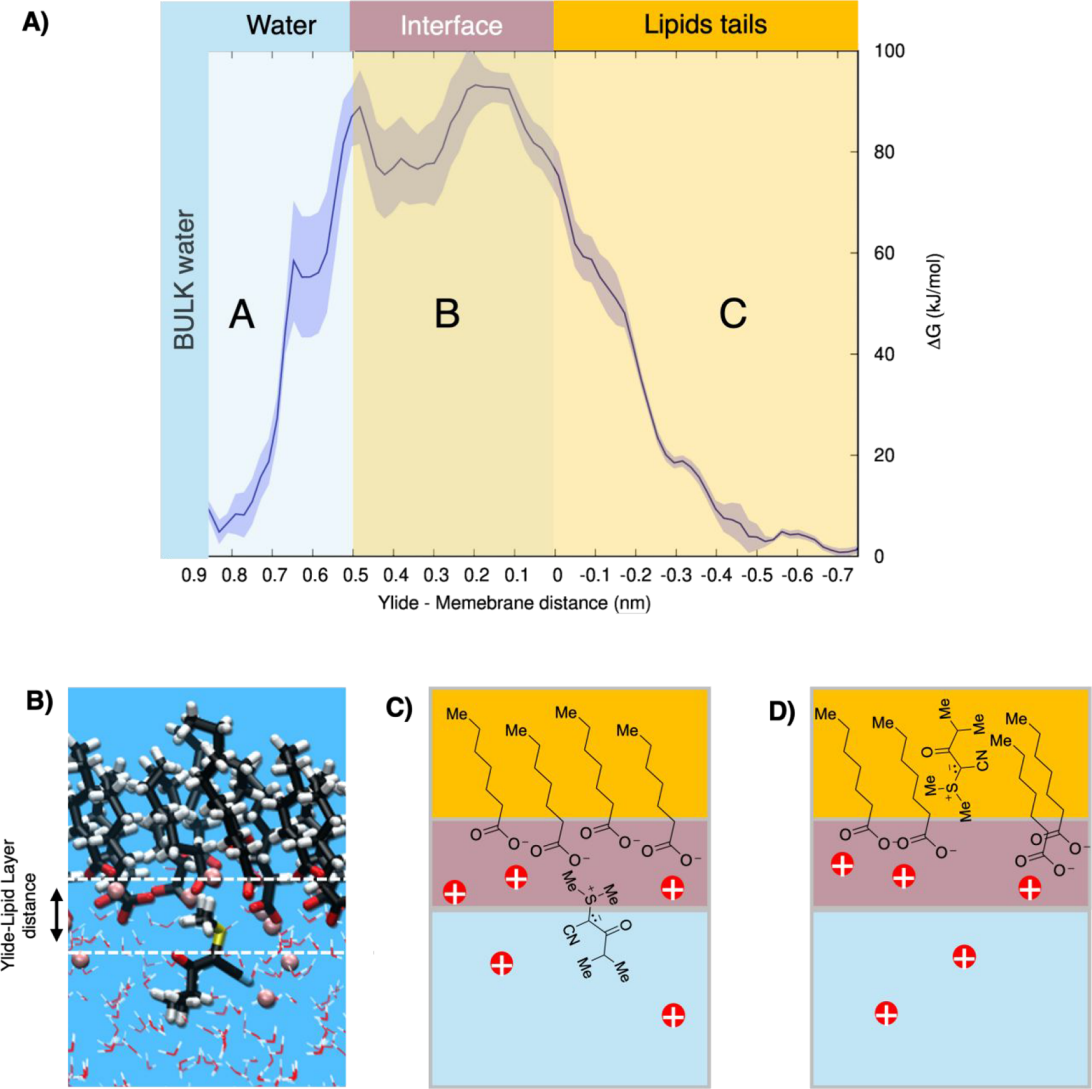
(A) DFTB-MD (eXtended Tight Binding GFN1-xTB) of ylide-lipid layer free energy profile (see computational section for details). (B) Illustration of the adopted definition Ylide-lipid heads distance reaction coordinate, i.e., the distance along *z* between the center of mass of the COO- heads of the heptanoates. (C) Orientation of the ylide residues when located at the interface and (D) within hydrophobic environment (zone C).

Using enhanced molecular dynamics simulations, we investigated the penetration of a single ylide residue into the membrane. The free energy profile along the ylide-surface distance clearly shows three zones where the molecule is stable (Figure 6A). The first zone (A) corresponds to the molecule fully solvated in the bulk water. This minimum was expected due to the molecule’s strong hydrophilic nature. The second zone (B) corresponds to the ylide in the interfacial region, with the molecule oriented to maximize the dipole–dipole interactions with the electric double layer (Figure 6C).

The positively charged sulfonium residue settles near the negatively charged membrane surface. Interestingly, the S^+^/COO^–^ radial pair distribution function obtained on the unbiased BLYP molecular dynamic simulations shows a large maximum between 2.8 and 5.0 Å, with two subpeaks at 3.6 and 4.3 Å (see the Supporting Information for more details). This suggests a structure oscillating between a contact and a solvent-separated ion pair. We speculate that the full contact ion pair situation is hindered by the steric bulk of the hydrophobic methyl residues and the repulsion between the negatively charged groups of the ylide and the carboxylate ions. Our observation aligns with studies on N-oxides conducted by Zhan and co-workers, which demonstrate that the salt-resistant behavior of N-oxides originates from electrostatic repulsion and can be directly correlated to the short distance between the positive and negative charges.^57^

Energetically, the ylide settling at the interface (zone B) is much less favorable than in bulk water (zone A). The dipole–dipole interactions that stabilize zone B only partially counterbalanced the loss of solvation energy and the repulsive interactions of the ylide negative poles and the heptanoic acid heads. However, the latter are partially screened by potassium cations. Still, the existence of zone B has important consequences, as we will see below. The third stable zone (C) corresponds to the ylide fully located inside the hydrophobic layer formed by alkyl chains, resembling the hydrophobic environment of a membrane. Again, the molecule tends to align with the direction that favorably maximizes the dipole–dipole interaction, i.e., with the sulfur pointing to the negatively charged carboxylate heads in this case (Figure 6D). However, here the interactions between the negatively charged pole of the ylide and the carboxylates are no longer screened by the potassium cations and by water. The molecule is pushed far into the membrane layer, and any direct interaction of sulfonium with the carboxylate is lost.

Interestingly, the ylide residue is energetically more stabilized in the hydrophobic environment in zone (C) compared with the interface of zone (B). Our BLYP calculations on unbiased trajectories (See section 7 of the SI for more details) indicate that the ylide can rearrange its electronic cloud in a way to reduce its dipole moment when a hydrophobic environment is present from 9.5 (±0.9) Debye in zone B, to 7.4 (±0.9) Debye in zone C, and to 5.5 (±0.5) Debye in vacuum, hence overcoming the loss of solvation energy. The barrier for crossing the carboxylates is quite asymmetric. Indeed, once the ylide is in the hydrophobic milieu, it can diffuse back into the solution. Notice that on the waterside, the potassium cations and the water can provisionally rearrange to provide a partial screening for the repulsive interaction with the negatively charged carboxylates, helping the ylide overcome the barrier and enter the membrane. The aid is lost inside the membrane. The obtained theoretical insights on the behavior of C-ylides at a hydrophilic and hydrophobic interface help to support the experimental results.

When the fully water-soluble poly(SY-AAm) is present in an aqueous solution, the higher stability of the polymer in bulk water (zone A) compared with the membrane interface (zone B) prevents the polymer from being near the membrane in relevant concentrations. However, when poly(SY-AAm) is present in the form of a coating, the effective concentration is increased, and the absence of bulk water will place poly(SY-AAm) at the interface (zone B). This is possible because zone B provides a thermodynamically stable minimum. From this position, the ylide is thermodynamically pushed inside the membrane (zone C), which is more stable than the location at the interface (zone B). Consequently, it induces membrane damage and cell death of bacterial cells that adhere to coated surfaces.

## Conclusions

The use of ylides to generate hydrophilic polymers has led to the development of new zwitterionic materials including antimicrobial coatings. To date, two types of polymeric ylides have been reported: N-oxide-based and C-ylide-based polymers.^15,17,23,25^ While significant attention has been devoted to elucidating the structure–property relationships of N-oxide-derived materials, studies on the mechanisms of C-ylides remain rare, despite their significantly broader chemical space. Here, we accessed a fully water-soluble acrylamide-based polymer bearing sulfur ylide residues, which is in stark contrast to previous reports in which sulfur ylides were placed on a hydrophobic polystyrene backbone. While surfaces that are coated with the water-soluble poly(ylide) displayed similar properties compared to surfaces treated with the styrene-derived poly(sulfur ylides)—most importantly inducing toxicity to bacterial cells—the water-soluble poly(sulfur ylide) lost its toxicity toward bacterial cells in solution. Previous studies on polymeric ylides have shown that styrene-derived poly(sulfur ylides) and poly(phosphorus ylides) did not exhibit cytotoxicity toward mammalian cells, indicating that their toxicity was primarily directed toward bacterial cells.^23,25^ Further investigations into various polymeric ylide scaffolds, including hydrophilic poly(ylides), will help determine whether this selectivity applies to all poly(ylides). Computational studies reveal that ylide residues are highly stabilized in bulk water, and tend to screen negatively charged surfaces. Importantly, we observe an environmentally responsive dipole moment, resulting in the stabilization of ylide residues within hydrophobic environments. Genetic analysis of both water-soluble and water-insoluble polymeric ylides revealed similar interactions with the outer membrane. For example, genes related to MlaA are overexpressed, indicating destabilization of the outer membrane to some extent, which confirms computational analysis. We speculate that in the case of the water-insoluble poly(sulfur ylide), the more hydrophobic polystyrene backbone provides additional driving forces to cause membrane interactions and disruption; in contrast, the polyacrylamide-derived poly(sulfur ylides) lacks this hydrophobicity. Additionally, the polystyrene-derived poly(sulfur ylide) displays strong amphiphilicity, likely resulting in defined supramolecular interactions and assemblies. In the future, we aim to reveal these interactions and further enhance our understanding of structure–property relationships. Interestingly, the polyacrylamide-derived poly(sulfur ylides) maintain their on-surface toxicity, likely caused by the absence of bulk water. Since our computational model only simulates a negatively charged interface, it will be interesting in future studies to elucidate its precise interactions with cell membranes, either experimentally, or computationally, by taking into account specific anionic residues typically displayed at extracellular membranes and differences between, for example, mammalian and bacterial membranes.

## Supporting Information Available

Experimental and computational details; characterization data (PDF)
