# Supplementary material for: Water-Soluble Sulfur-Ylide-Functionalized Polyacrylamides for Antibacterial Surface Applications
Source: Langmuir. 2025 Mar 24;41(13):8627–36. doi: 10.1021/acs.langmuir.4c05134 (PMC11984111; doi:10.1021/acs.langmuir.4c05134)
Supplement: Supplementary file 1 — la4c05134_si_001.pdf [file la4c05134_si_001.pdf]

Water-Soluble Sulfur-Ylide Functionalized Polyacrylamides for Antibacterial Surface Applications

Bela B. Berking[‡a], Dimitrios Karagrigoriou[‡a], Daria R. Galimberti[*b], Bai H. E. Zhang[a], Daniela A. Wilson[*a], Kevin Neumann[*a]

[a]Systems Chemistry Department, Institute for Molecules and Materials, Radboud University, Heyendaalseweg 135, 6525 AJ Nijmegen, The Netherlands

[b] Theoretical and Computational Chemistry Department, Institute for Molecules and Materials, Radboud University Nijmegen, Heyendaalseweg 135, 6525 AJ Nijmegen, The Netherlands

E-mail: daria.galimberti@ru.nl, d.wilson@science.ru.nl, kevin.neumann@ru.nl

Table of Content

1. Experimental
2. Supporting Figures
3. Experimental Protocols
4. Surface Energy Analysis
5. Surface Immobilization
6. Biological Assays
7. Computational Calculations
8. NMR Spectra
9. References

1. Experimental

1.1 Materials

Reagents were obtained from Sigma Aldrich/Merck (Zwijndrecht, The Netherlands), Fluorochem BV (Amsterdam, The Netherlands) and TCI Europe (Zwijndrecht, Belgium) and were used without purification unless otherwise stated. Boc-Inp-OH was obtained by fluorochem. Solvents were obtained from VWR, Fisher, Acros Organic and Sigma Aldrich/Merck. Solvents were dried by passing over activated alumina columns in a MBraun MB SPS800 under a nitrogen atmosphere and stored under argon.

Reactions were carried under air unless stated otherwise. Typically, such air-sensitive reactions were carried out under atmosphere of nitrogen using Schlenk technique. Ultrapure Milli-Q water was obtained from QPOD Milli-Q system. Reactions and fractions from flash column chromatography were monitored by thin layer chromatography using glass TLC plates (Merck, TLC Silica gel 60 F_{254}) and if necessary visualized by staining with $KMnO_4$ solution. Column chromatography was performed on VWR SiO_2 Type (40-63 mesh) using a forced flow of air at 0.5-1.0 bar. The poly(SY-Sty) and polystyrene used in this study, was reported previously by our group. The same polymer was used without any modifications.[1] 5 kDa PEG-COOH (PSB-227) was obtained from creative PEGWorks.

1.2 Instrumentation

Gel permeation chromatography (GPC) equipped with PL gel 5 µm mixed D column calibrated for polystyrene (580– 377400 g/mol) was carried out on a Shimadzu instrument with NMP as eluent using differential refractive index and UV absorbance (254 nm). SEC was run in water/acetonitrile mixtures on BioSep-SEC-s2000 column (300 x 7.8 mm).

2. Supporting Figures

2.1 Synthetic schemes

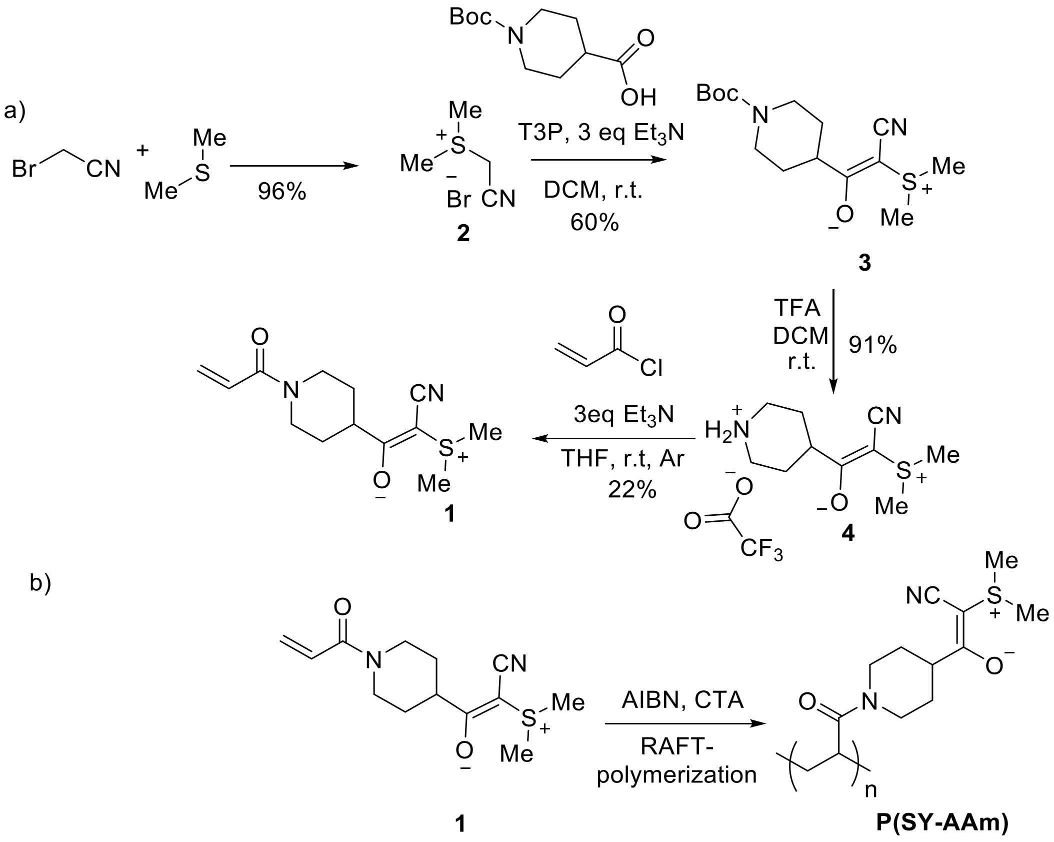

Figure S1. Synthetic route towards Poly(SY-AAm) with **a)** synthesis of the monomer **1** from bromoacetonitrile and dimethyl sulfide and **b)** polymerization of the monomer **1** using RAFT polymerization for the synthesis of the homopolymer Poly(SY-AAm).

2.2 Solubility

Solubility of poly(SY-AAm) was tested in various solvents at room temperature by dissolving 1 mg of the polymer in 1 mL of solvent. The mixtures were vortexed for a while and the solubility was determined by visual appearance. If the polymer did not dissolve, then it was sonicated for 10 min with heating and then the solubility was again checked.

Table S1. Solubility of poly(SY-AAm) in different solvents, green = soluble (> 1 mg/mL), yellow = partly soluble (1 mg/mL > solubility > 0 mg/mL), red = insoluble (no solubility observed).

**Polymer**	Water	THF	Et_2O	DMSO	CH_2Cl_2	Toluene	CH_3CN	MeOH	Acetic acid
P(SY-AAm)									

2.3 DSC

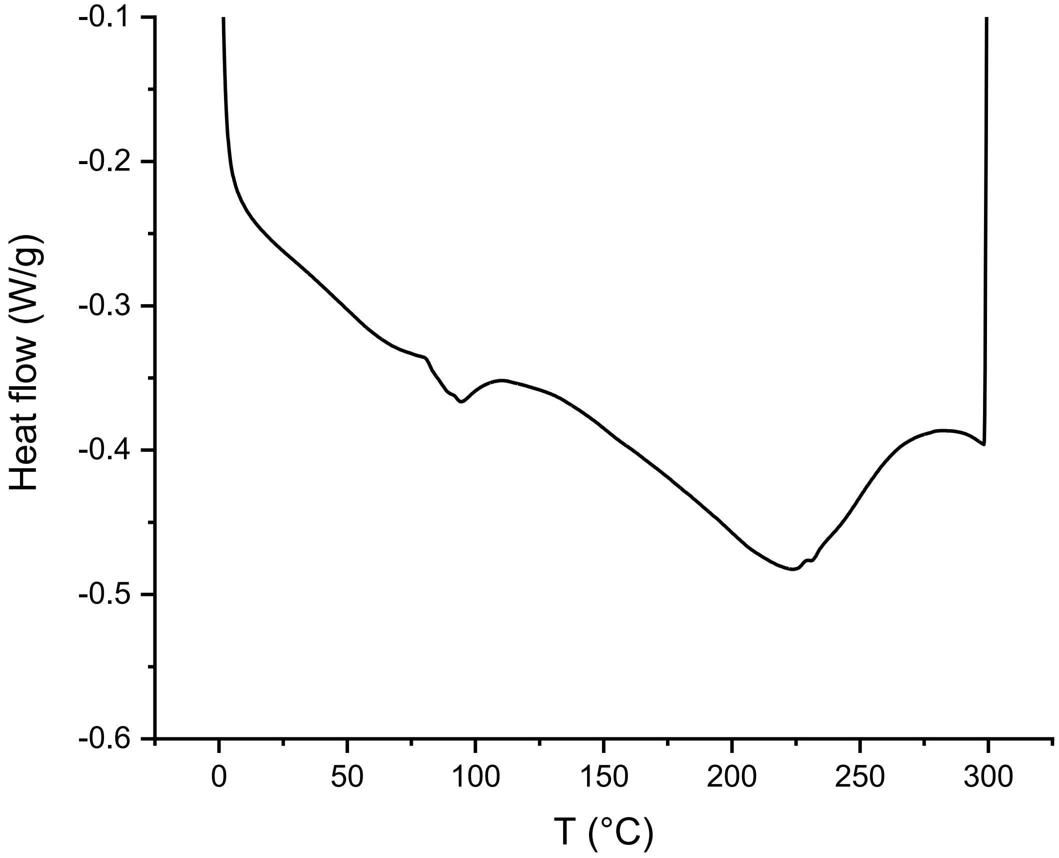

Figure S2. DSC traces of poly(SY-AAm)

3. Experimental protocols

3.1 Small molecules synthesis

Sulfonium salt 2

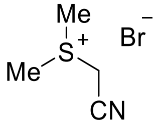

The synthesis of the sulfonium salt **2** was performed according to a protocol from literature.[2] Bromoacetonitrile (5.6 mL. 80 mmol, 1.0 eq) was placed in a flame dried and Argon flashed Schlenk-flask and dimethylsulfide (6.0 mL, 80 mmol, 1.0 eq) was added slowly. The solution was stirred overnight under Ar. The obtained white solid was washed several times with diethylether and dried under reduced pressure (yield 96 %).

^{1}H NMR (400 MHz, Dimethyl sulfoxide -d_6) δ 4.99 (s, 2H), 3.07 (s, 6H).

Boc protected acrylamide sulfur ylide precursor 3

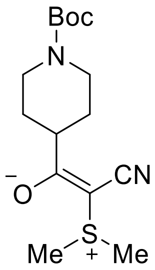

Boc-Inp-OH (4.5 g, 20 mmol, 1.0 eq) was dissolved in dichloromethane (120 mL) and the mixture was stirred. Triethylamine (8.3 mL, 59 mmol, 3.0 eq) was added followed by T3P (15.2 mL, 25.5 mmol, 1.3 eq). The mixture was stirred for 10 min and then sulfonium salt **2** (5.0 g, 28 mmol, 1.4 eq) was added. The reaction mixture was stirred at room temperature overnight. It was then diluted with dichloromethane and it was washed with sat. $NaHCO_3$ (x1), H_2O (x1) and brine (x1). The crude product was dried over Na_2SO_4 and purified via column chromatography on silica gel eluting with CH_2Cl_2/MeOH mixtures (gradient to 94:6 to 90:10 by volume) to obtain the product **3** as a brown solid (yield 60 %).

^{1}H NMR (400 MHz, Chloroform-*d*) δ 4.31 – 3.91 (m, 2H), 2.85 (s, 6H), 2.83 – 2.80 (m, 1H), 2.80 – 2.68 (m, 2H), 1.77 – 1.68 (m, 2H), 1.68 – 1.53 (m, 2H), 1.45 (s, 9H). ^{13}C NMR (101 MHz, Chloroform-

d) δ 194.2, 154.7, 119.2, 79.4, 52.7, 44.8, 43.0, 28.4, 28.3, 28.2. HRMS (ESI): calculated for $[M+H]^+$: m/z 335.14053, found: m/z 335.13993

Deprotected acrylamide sulfur ylide precursor 4

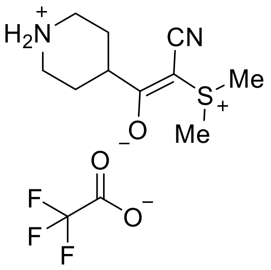

Compound **3** (3.5 g, 11 mmol, 1.0 eq) was dissolved in dichloromethane (14 mL) and then trifluoroacetic acid (14 mL) was added dropwise and the reaction was stirred at room temperature for 2 h. Then, it was precipitated in Et_2O to obtain the product as a light brown solid. The solid was isolated by filtration under vacuum and washed with cold Et_2O (yield 91 %).

^1H NMR (400 MHz, Deuterium Oxide) δ 3.46 – 3.38 (m, 2H), 3.07 – 2.93 (m, 4H), 2.77 (s, 6H), 1.97 – 1.88 (m, 2H), 1.83 – 1.72 (m, 3H). ^{13}C NMR (101 MHz, Deuterium Oxide) δ 194.0, 163.1, 162.7, 120.7, 119.0, 117.8, 114.9, 112.0, 60.6, 43.2, 41.6, 27.6, 24.9. HRMS (ESI): calculated for $[M]^+$ (cation): m/z 213.10616, found: m/z 213.10323.

Acrylamide sulfur ylide monomer 1

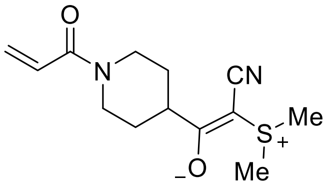

Acryloyl chloride (0.38 mL, 4.6 mmol, 1.5 eq)) was dissolved in dry tetrahydrofuran (15 mL) in a flame dried and argon flushed Schlenk-flask in ice bath. Then trimethylamine (1.3 mL, 9.2 mmol, 3.0 eq) and the sulfur ylide **4** (1.0 g, 3.1 mmol, 1.0 eq) were added quickly in the flask in ice bath. Then the ice bath was removed, and the reaction mixture was stirred at room temperature under argon overnight. It was then diluted with dichloromethane and washed with $NaHCO_3$ (X1), H_2O (X1) and brine (X1). The crude product was dried over Na_2SO_4 and purified via column

chromatography on silica gel eluting with CH_2Cl_2/MeOH mixtures (gradient 96:4 to 90:10 by volume) to obtain the product 1 as a yellow solid (yield 22 %).

^{1}H NMR (400 MHz, Chloroform-*d*) δ 6.58 (dd, *J* = 16.8, 10.6 Hz, 1H), 6.22 (dd, *J* = 16.9, 2.0 Hz, 1H), 5.66 (dd, *J* = 10.6, 2.0 Hz, 1H), 4.69 – 4.61 (m, 1H), 4.07 – 4.00 (m, 1H), 3.19 – 3.07 (m, 1H), 3.00 – 2.88 (m, 1H), 2.85 (s, 6H), 2.80 – 2.68 (m, 1H), 1.88 – 1.76 (m, 2H), 1.75 – 1.52 (m, 2H). ^{13}C NMR (101 MHz, Chloroform-*d*) δ 193.5, 165.4, 128.0, 127.3, 119.2, 52.7, 45.5, 44.5, 41.7, 28.7, 28.3. HRMS (ESI): calculated for $[M+Na]^+$: m/z 289.09867, found: m/z 289.09587.

3.2 Poly(acrylamide sulfur ylide) (poly(SY-AAm)) characterization

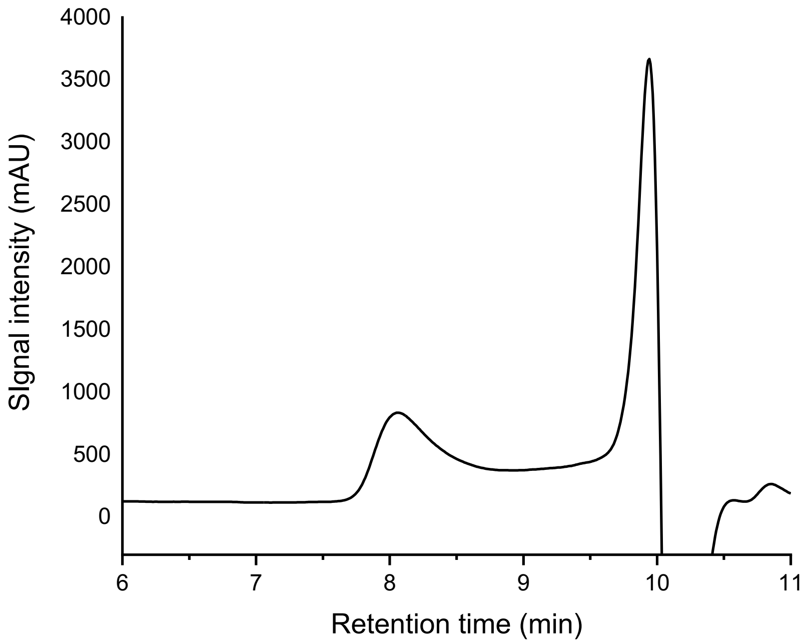

Figure S3. GPC (NMP, polystyrene calibration): M_n= 2.1 × 10^3 g/mol, M_w/M_n = 1.04

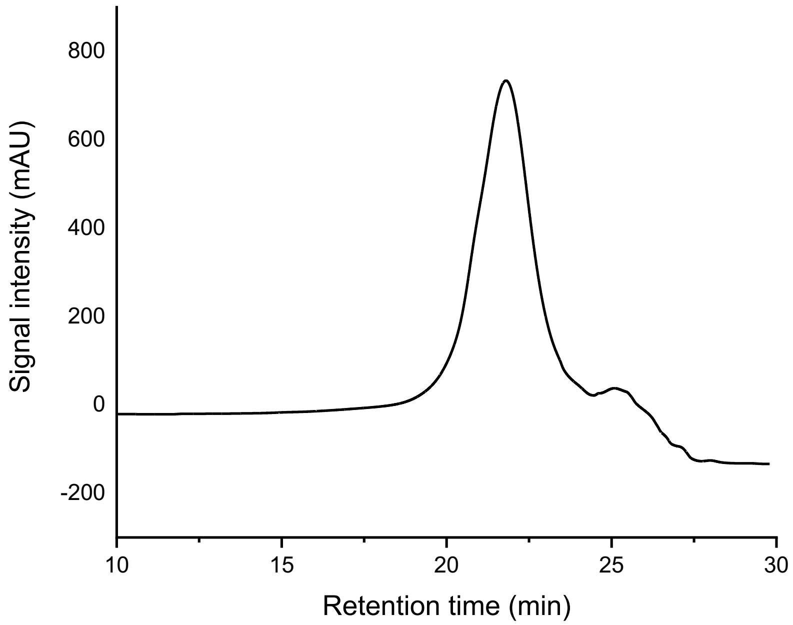

Figure S4. SEC (50% MilliQ, 50% acetonitrile).

^{1}H-NMR (500 MHz, Dimethyl sulfoxide-d_6) δ(ppm): 4.72–3.48 (br. m, 2H (H_{3eq}, H_{4eq})), 3.22–2.30 (br. m, 4H (H_1, H_{3ax}, H_{4ax}, H_7)), 2.86–2.75 (br. s, 6H (H_8)), 1.90–0.90 (br. m, 6H (H_2, H_5, H_6)).
M_n (NMR) = 6.4×10^3 g/mol.

The molecular weight of polymers was determined *via* ^{1}H-NMR using the proton adjacent to the dithioester.

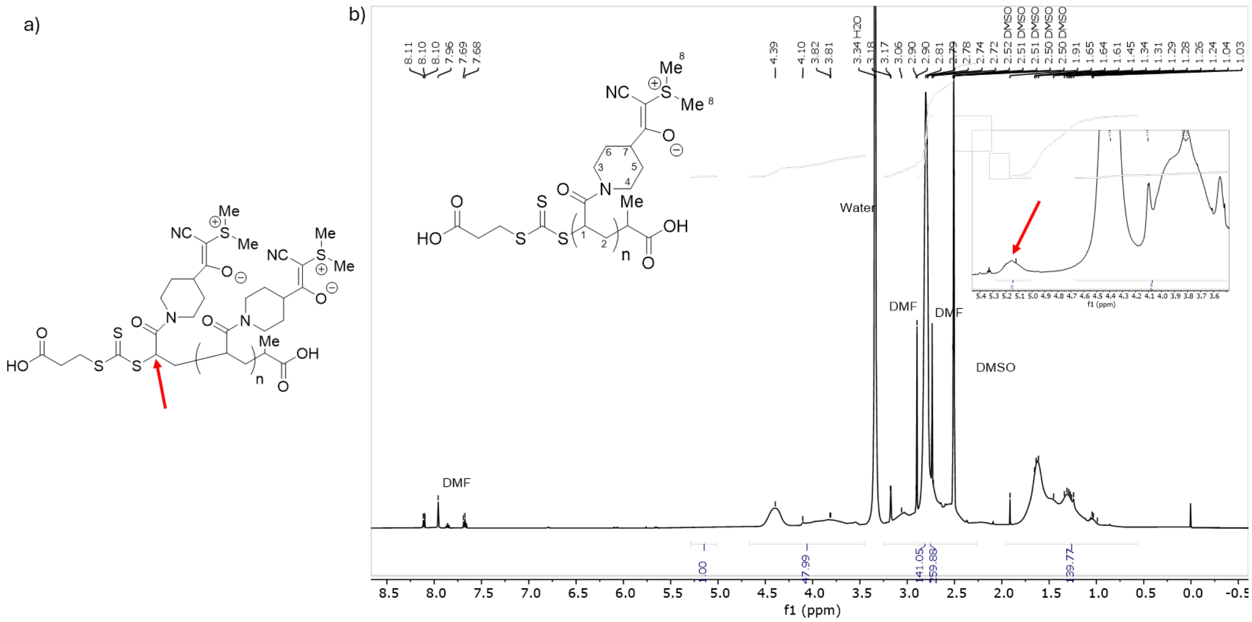

Figure S5. M_n and X_n determination of the Poly(SY-AAm): a) Poly(SY-AAm) structure (the protons used as reference are indicated with an arrow), b) ^1H NMR spectrum of Poly(SY-AAm) and inset ^1H NMR spectrum showing the proton signals used as reference.

4. Surface energy analysis

4.1 Covalent attachment

Poly(SY-AAm) was dissolved in water and EDC (3 eq) and NHS (5 eq) were added. The polymer solution was placed on an amine-coated glass surface in a silicone cylinder and left overnight in the fridge at 4 ^{o}C. Then, the silicone cylinder was removed and the surface was rinsed with MIlliQ, EtOH, EtOH/MilliQ and MIlliQ, and subsequently dried by air and with a 'super-soft tissue'.

4.2 Contact angle measurements

For the contact angle measurements, MiliQ water, glycerol or diidomethane (3 µL) was placed on the coated polymer surface and then snapshots were taken using optical microscopy. The images were then analyzed using *imagej* program to determine the contact angles. After each measurement, the surface was cleaned with MilliQ and dried with a 'super soft tissue'. Each measurement was performed four times.

The surface energy was calculated using the acid-base Van Oss method.[3]

1) $0.5\,\gamma_L\,(1 + cos\Theta) = \sqrt{\gamma_S^{LW}\gamma_L^{LW}} + \sqrt{\gamma_S^{+}\gamma_L^{-}} + \sqrt{\gamma_S^{-}\gamma_L^{+}}$

2) $\gamma_S = \gamma_S^{LW} + 2\sqrt{\gamma_S^{+}\gamma_S^{-}}$

Θ: contact angle; γ_S: (total) surface energy of a polymer; γ^{LW}: the Liftshitz/van der Waals (dispersive) component; γ^{+}: Lewis acid component; γ^{-}: Lewis-base component; γ_L: surface tension of the liquid; mN/m (or mJ/cm^{2}); L = liquid, S = solid.

Table S2. The values of γ_L^{LW}, γ_L^{+}, γ_L^{-} and γ_L for water, glycerol and diiodomethane found from literature.[4,5]

	**γ_L (mN/m^2)**	**γ_L^{LW} (mN/m^2)**	**γ_L^{+} (mN/m^2)**	**γ_L^{-} (mN/m^2)**
Water (MilliQ)	72.8	21.8	25.5	25.5
Glycerol	64.0	34.0	3.92	57.4
Diiodomethane	50.8	50.8	0	0

To find γ_S^{LW}, γ_S^{+} and γ_S^{-}, equation **1** was solved using the contact angles measured (table S3) and the constants of table **S2**. Then γ_S was calculated using equation **2**.

Table S3. The contact angles measured for the Poly(SY-AAm).

**Polymer**	**MillliQ Water**	**Diiodomethane**	**Glycerol**
P(SY-AAm)	42.1 ± 3.5	40.6 ± 1.2	48.6 ± 3.6

5. Surface attachment of polymers

For surface attachment to well plates, amine-coated well plates were used. The well plates were commercially available and were obtained from biomat (MCB02F-AM1). Well-plates were modified with polymers that bear carboxylic acids using the following protocol: carboxylic acid containing polymers (poly(SY-AAm), polystyrene and PEG) were dissolved in miliQ water (10 mM) and EDC (3 equiv) with NHS (5 equiv) were added. Polystyrene was dissolved in THF (10 mM). The solution was settled for 10 min and added to the well plates. After 4 hours, the solution was removed and the wells were thoroughly washed with EtOH, water/EtOH, water and finally with EtOH again to guarantee rapid drying. Finally, the well-plates were air-dried overnight. In a similar manner, amine-coated glass slides were modified. This was achieved by using a deep well (2 x 1 x 0.5 cm) and repeated the protocol established for the well plates. Because of the autofluorescence of polymeric sulfur ylides, it was possible to confirm the modification by measuring fluorescence (λ_{ex} = 380 nm, λ_{em} = 450 nm, bandwidth 20 nm).

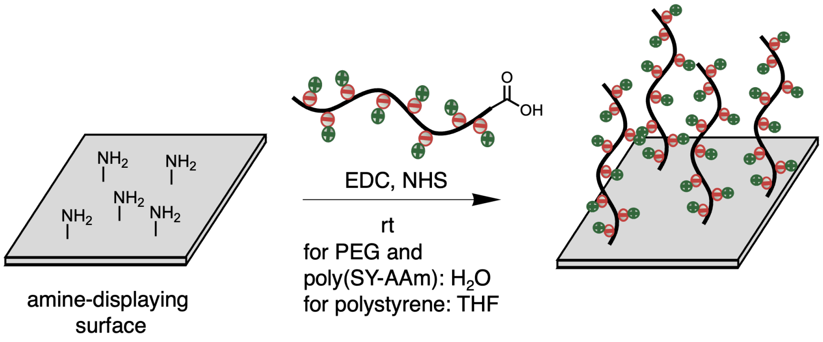

Figure S6. Chemistry employed for the coating of amine-displaying surfaces.

6. Biofilm Bacterial Analysis

Biofilm preparation

The Overnight culture was inoculated in 6 mL of Brain Heart Infusion (BHI) by adding 5 µL of *P. aeruginosa* ATCC 10145, 50% Glycerol Stock and incubated overnight at 37 °C. The next day, the resulting culture was diluted to an OD of 0.01 and was seeded to allow for biofilms to grow. The plates were incubated at 37 °C.

Confocal Microscopy

Various coated slides were prepared to assess biofilm growth. For this, silicon culture inserts were reversibly mounted on the slides, creating a reservoir which allowed for biofilm growth. Previously prepared bacterial solution diluted to an OD of 0.1 was then seeded into these reservoirs and left to incubate for 24 hours. Afterwards the biofilms were cleaned with PBS before being stained with Live Dead stain. Syto9 and Propidium Iodide were used to create a suitable working solution: for Syto9 a final concentration of c = 11.1 nM and for Propidium Iodide a final concentration of c = 66.6 nM in PBS (150 mM NaCl, 100 mM Na_3PO_4 mM, pH 7.4). Biofilms were stained for 10 minutes and washed three times with PBS (150 mM NaCl, 100 mM Na_2HPO_4, pH 7.4). The silicone culture inserts were then removed, and a coverslip glued over the biofilm populated areas with instant glue. Imaging was conducted using an SP8x AOBS-WLL confocal laser scanning microscope. Lasers were set at λ_{ex} = 470 nm, λ_{em} = 500-520 nm and λ_{ex} = 560 nm, λ_{em} = 620-670 nm. Images were later analyzed with Imaris.

Live/Dead Assay

96 well plates with various modified surfaces were inoculated with 100 µl of bacterial solution in BHI broth (OD 0.005) and incubated for 4 hours at 37 °C to allow for adhesion and biofilm formation. After 4 hours, all wells were gently washed three times with 1x PBS buffer (pH 7.4) to remove planktonic cells. BacLight stain (Molecular Probles) containing Syto9 and Propidium

Iodide was used to create a suitable working solution: for Syto9 a final concentration of c = 11.1 nM and for Propidium Iodide a final concentration of c = 66.6 nM in PBS (150 mM NaCl, 100 mM $NaPO_4$ mM, pH 7.4). Wells were stained for 10 minutes and washed three times with PBS (150 mM NaCl, 100 mM $NaHPO_4$, pH 7.4). Fluorescence intensity was measured at λ_{ex} = 485 nm, λ_{em} = 535 nm and λ_{ex} = 300 nm, λ_{em} = 632 nm, respectively, with a bandwidth of 20 nm, 30 flashes and an Integration time of 40 µs using a Tecan Spark M10 plate reader.

Crystal Violet Stain

96 well plates with various modified surfaces were inoculated with 100 µl of bacterial solution in BHI broth (OD 0.005) and incubated for 4 hours at 37 °C to allow for adhesion and biofilm formation. After 4 hours, all wells were gently washed three times with 1x PBS buffer (pH 7.4) to remove planktonic cells and subsequently stained for 10 minutes with 0.01 % crystal violet in water (w/v). CV solution was then removed, and wells washed three times with PBS buffer before letting the well plate dry overnight fpr analysis. Stained biomass was resolubilized in 30% acetic acid in distilled water (v/v) and transferred to a new clear bottom well plate. Absorbance was measured at 590 nm in a Tecan Spark M10 plate reader.

Cytotoxocity assay

An overnight culture was prepared as described above with the addition of 0.04 % Tyloxapol. After 24 hours bacteria were adjusted to an OD of 0.01 in various concentrations of P(AmSY). 100 µl of bacterial-polymer solution were added into 96 well plates and left to incubate for 24 hours at 37°C. Afterwards the OD was measured using a Tecan Spark M10 platereader at 600 nm. Experiments were carried out in replicates of n= 7-9.

RNA Extraction

Bacteria harvested from an overnight culture were diluted to an OD of 0.1 in the different treatments (0.1 mg polymer/mL) all prepared in BHI broth and left to incubate at 37°C for 4 hours. The total RNA was extracted from the biofilms using the RNeasy Kit of QIAGEN. Bacteria were spun down and pellets resuspended in 1x PBS and RNA protect reagent in a 1 to 2 ration. The samples were transferred into bead-beating tubes and the cells were lysed by 0.1mm Zirconia/silica beads in the BeadBug 6 bead homogenizer for 3 cycles of 30 seconds on and off at 4000rpm. Following the lysis, the lysate was transferred to the RNeasy Mini Spin Column and placed in 2 mL collection tubes. The columns were centrifuged at ≥8000x g for 15 sec. and the flow-through was discarded and the collection tube was reused. This step was repeated until all lysate was processed. Then to wash the spin column membrane 700µl Buffer RW1 was added and centrifuged at ≥8000x g for 15 sec. The flow-through was discarded and the columns were placed in new collection tubes. Subsequently, 500µl Buffer RPE was added and centrifuged at ≥8000x g for 15 sec. This step was repeated once more with 500µl Buffer RPE and centrifuged at ≥8000x g for 2 min. to ensure the removal of ethanol. The spin columns were then transferred to new 1.5 ml collection tubes, and 30-50µl of Rnase-free water was added directly to the membrane. The columns were then centrifuged at ≥8000x g for 1 min. to elute the RNA. The concentration and purity of the RNA were determined by the Nanodrop 1000™ spectrophotometer. Additionally, an Agarose Gel electrophoresis was conducted to verify presence of 16s RNA.

cDNA Synthesis

For the cDNA synthesis, the RNA was treated with DNase to remove genomic DNA. This was done using the DNase I Amplification Grade by Invitrogen™. The following was added to an RNase-free PCR-strips on ice: 500 ng total RNA, 1µl 10X DNase I Reaction Buffer, 1µL DNase I Amplification Grade (1U/µl) and DEPC-treated water to 10µL. Then the PCR strips were incubated for 15 min. at room temperature. Then 1µl of 25 mM EDTA was added to inactivate the DNase I. Lastly, the RNA samples were incubated for 10 min. at 65°C. cDNA was synthesized using the SuperScript™

II Reverse Transcriptase by Invitrogen™. To each DNase-treated RNA 9µl of the following mix were added: 1µl random primers (250 ng/µl), 1µl 10mM dNTP's, 4µl 5X 1st Strand Buffer, 1µl 0.1M DTT, 1µl RNaseOUT™ (10 U/µl), 0.5µl Superscript II (200 U/µl) and 0.5µl DEPC-treated water. Mixed and incubated for 10 min. at 25°C followed by another incubation of 50 min. at 42°C and the reaction was inactivated by heating it for 15 min. at 70°C. After the cDNA synthesis, the cDNA was purified using the QIAquick PCR Purification Kit of QIAGEN. This was done according to the manufacturer's instructions. Following purification, the cDNA concentration was measured with the Qubit™ 4 Fluorometer using the 1X High Sensitivity dsDNA assay.

qRT-PCR

qRT-PCR was performed using the iQ SYBR Green Supermix by Bio-Rad. Each reaction contains 10µL iQ SYBR Green Supermix, 2µL (10µM) forward primer, 2µL (10µM) reverse primer, 1ng cDNA and DEPC-treated water to a final volume of 20µL. The primers that were used are listed in Table S4.

The qRT-PCR was performed in a Bio-Rad C1000 Touch Thermal Cycler using the following protocol: 95°C for 30 seconds, 60°C for 10 seconds and 72°C for 20 seconds (repeat 39X). Followed by a melt-curve analysis from 58°C to 95°C at a 0.5°C/cycle melt rate. The relative gene expression was calculated using the 2-ΔΔCT method, where all Ct values were normalized to the housekeeping genes gyrA and recA. All samples were carried out in replicates of n=3.

Table S4. Primer design for all genes of interest. Primers were designed using Primer3 and verified via Blast.

**Gene of Interest**	Forward Sequence (5'->3')	Reverse Sequence (5'->3')
MlaA	GCATCAACCGTCCCATCTTC	GGTTGTTGGCCAGGTTCTTC
sigX	CAAGCGCCGATTGATGGATG	TCAACCTTCGGCGACTTCTC
pqsR	CCAATTACCGGCAGATCAGC	TCGTAGAGTTCGCTGAGGAC
recA	GAAGTTCTACGCCTCGGTCC	GTTCTTCACCACCTTGACGC
gyrA	ATGGAGGTGATCCGTGAGGA	TTCTTCACGGTACCGAAGGC

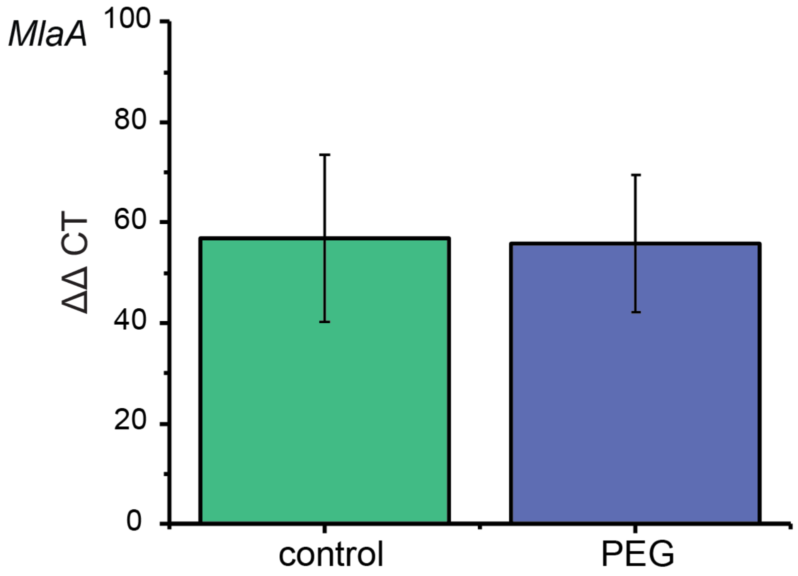

Figure S7. qRT-PCR analysis of genes MlaA upon incubation with PEG.

7. Computational

Additional computational details for the metadynamics simulation

As a first step of our simulation, we constructed the water-membrane interface. We started with a simulation box with dimensions of 15.6 Å x 15.6 Å x 52.0 Å, comprising the water solution and the heptanoic acids but not the ylide. We equilibrated it for 80 ps. in the NPT_F ensemble, i.e., constant temperature and pressure, using a flexible simulation box. In particular, the simulation box was allowed to relax and change shape along the x and y directions, but we fixed it along the z to maintain the vertical vacuum space, i.e., the a, b, and γ cell parameters were free to change, while c, a, and b were kept fix. For all the simulations, the average temperature of the system was set to 300 K using a CSVR thermostat with a time damping constant of 300 fs. The average pressure along x and y was set at 1 atm using a barostat with a time damping constant of 1 ps. The final cell parameters were a = 12.445, b = 15.707, c = 52.000, α = 90.0, β = 90.0, γ = 108.6.

As a second step, the ylide was inserted in the center of the water slab, and the system was equilibrated again. Starting from the equilibrated state, we have run an OPES metadynamics[6] still in the NPT_F ensemble, i.e., the membrane can open up to accommodate the ylide molecule. We selected the distance along z between the center of mass of the (C)OO^- heads of the heptanoic carboxylates and the center of mass of the ylide molecules as reaction coordinates. The initial guess for the barrier to overcome was 100 kJ/mol. The frequency for the kernel deposition was set to 200 steps. Two walls were placed at -7.5 Å (membrane side) and 14.5 Å (water solution side) to restrain the exploration of the molecule around the interfacial region. The metadynamics was run for a total simulation time of around 1.5 ns.

As a final sanity check, we run a short standard metadynamics simulation of 240 ps starting from the same equilibrated state and using the same collective variable as the OPES metadynamics. The frequency for the hills deposition was set to every 200 steps, the width of the Gaussian hills was set to 0.1, and the height to 0.5 kJ/mol. The final (qualitative) free energy profile (Figure S6) shows the same three minima obtained by the better converged OPES metadynamics.

Determination of ylide dipole

As described in the manuscript, six initial states (atomic positions, velocities, and cell parameters) were extracted from the metadynamics simulations: two with the molecule inside the membrane and four with the molecule in the interfacial minimum on the waterside. A set of unbiased BLYP DFT-MD simulations was run starting from these initial states (see the computational section of the main manuscript for more details). To evaluate the effect of the environment on the dipole moment of the ylide molecule, we extracted six shots from each of the unbiased BLYP MD simulations run at the end of the. For each snapshot, we computed the set of maximally localized Wannier functions[7] and corresponding Wannier centers. Using these letters, we estimated the dipole moment of the ylide molecule either inside the water solution or the membrane.

In addition, the gas phase dipole moment of the ylide has been estimated using a similar approach, employing a 20 ps BLYP DFT-MD (Density Functional Theory Molecular Dynamics) trajectory. This simulation was conducted with the ylide molecule placed in a box measuring 20.0 Å x 20.0 Å x 20.0 Å, following the same computational setup used for the other simulations.

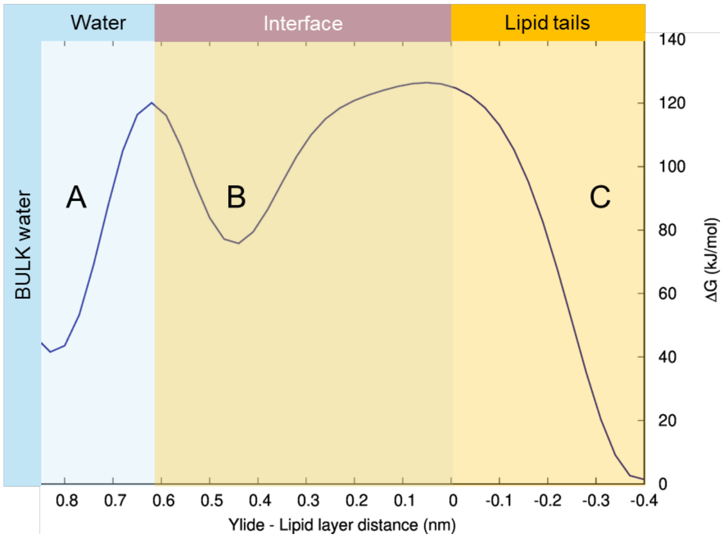

Figure S8. Free energy profile ylide being placed at the interface.

S^+/(C)OO$^-$ radial pair distribution function

In Figure S7, we reported the S^+/(C)OO$^-$ radial pair distribution function obtained for the two stable and unbiased BLYP molecular dynamic simulations at the water membrane interface (ZONE B) of the free energy profile (total simulation time 75ps). It shows a large maximum between 2.8 Å and 5.0 Å (Figure S7, yellow area), with two sub-peaks at 3.6 Å and 4.3 Å. This suggests a structure oscillating between a contact and a solvent-separated ion pair.

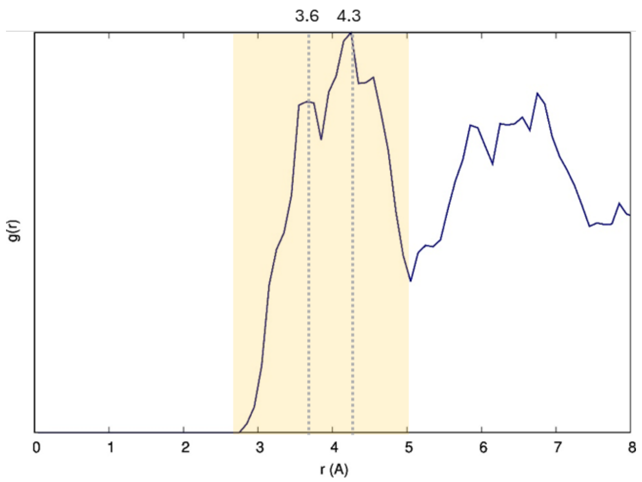

Figure S9. S^+/(C)OO$^-$ radial pair distribution function

8. NMR spectra

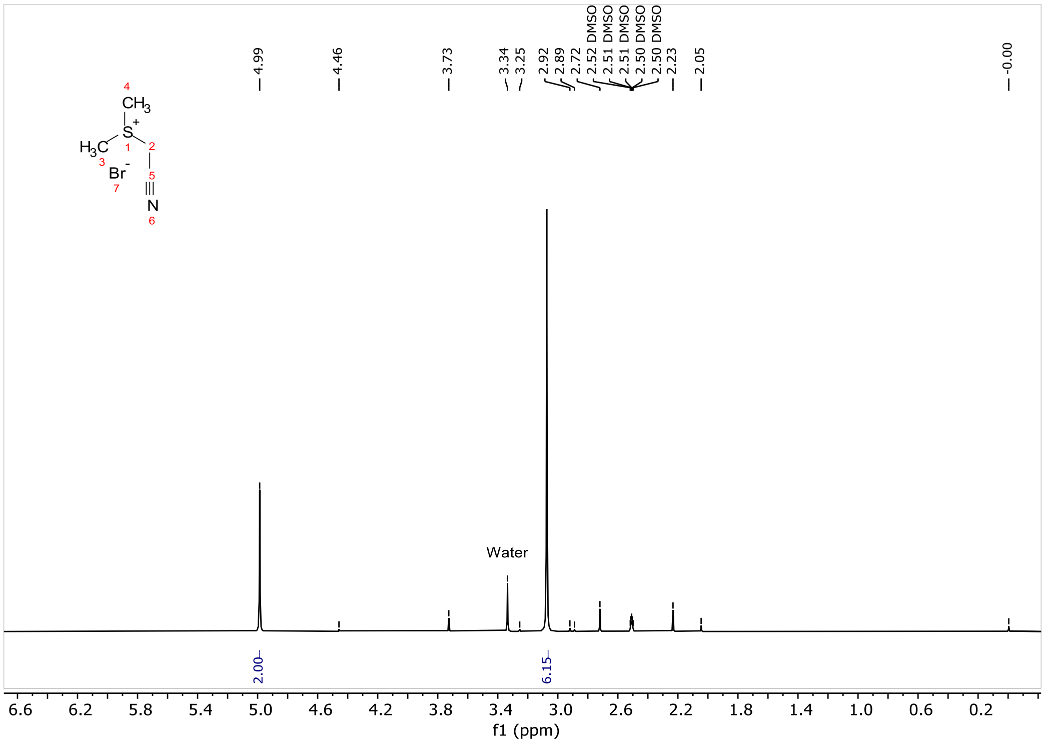

Figure S10. ^{1}H NMR spectrum (400 MHz, Dimethyl sulfoxide-d_6) of the sulfonium salt **2**.

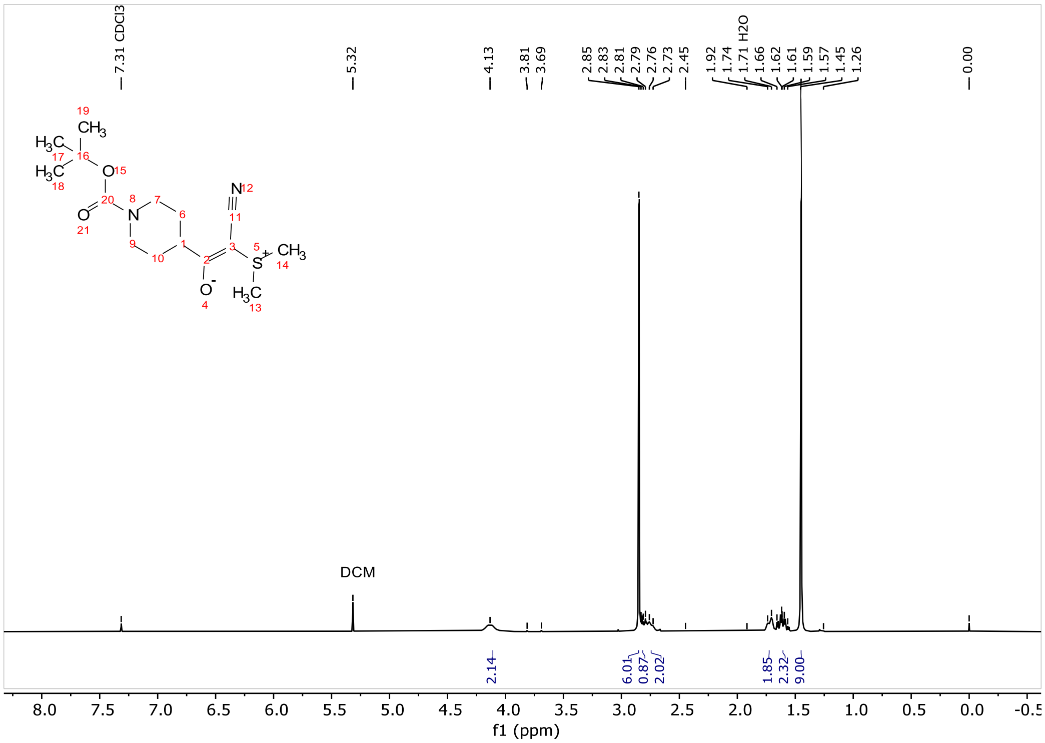

Figure S11. ^{1}H NMR spectrum (400 MHz, Chloroform-d) of the Boc protected acrylamide sulfur ylide precursor **3**.

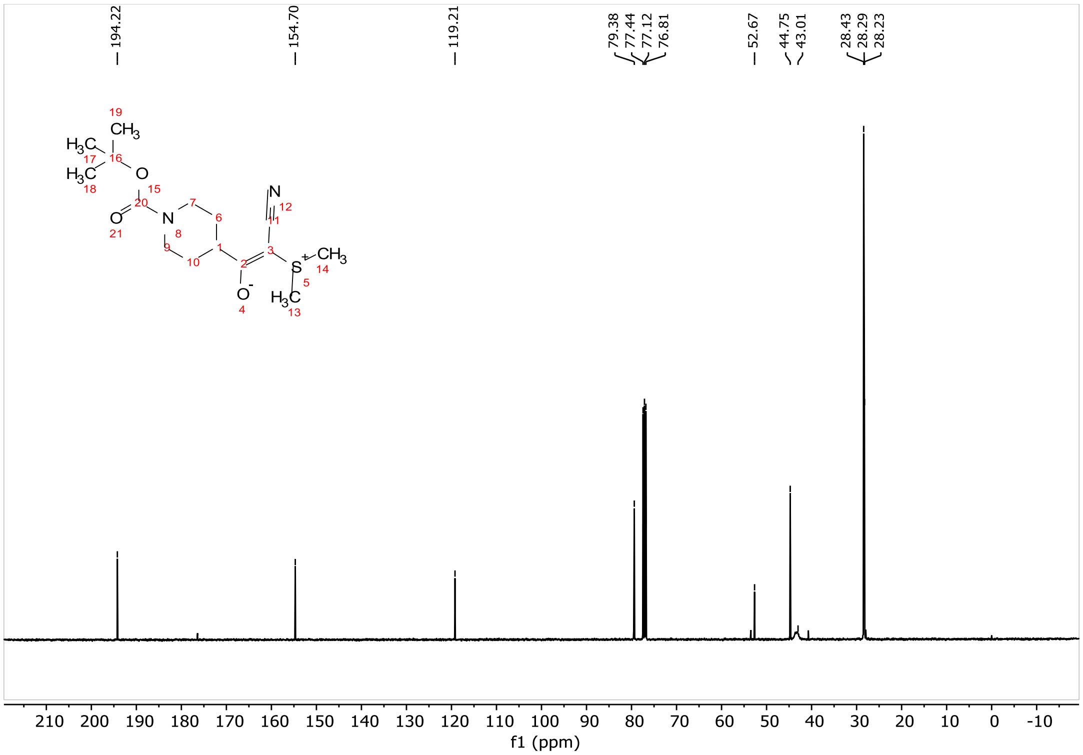

Figure S12. ^{13}C NMR spectrum (101 MHz, Chloroform-*d*) of the Boc protected acrylamide sulfur ylide precursor **3**.

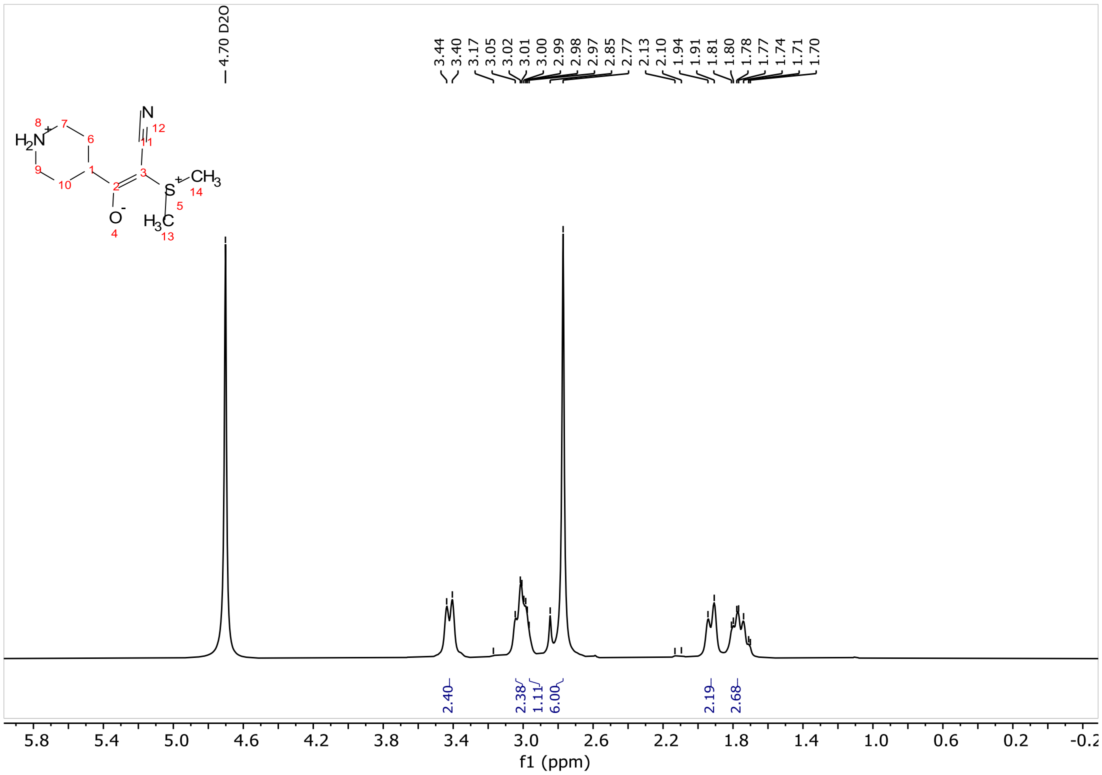

Figure S13. ^{1}H NMR spectrum (400 MHz, Deuterium Oxide) of the deprotected acrylamide sulfur ylide precursor **4.**

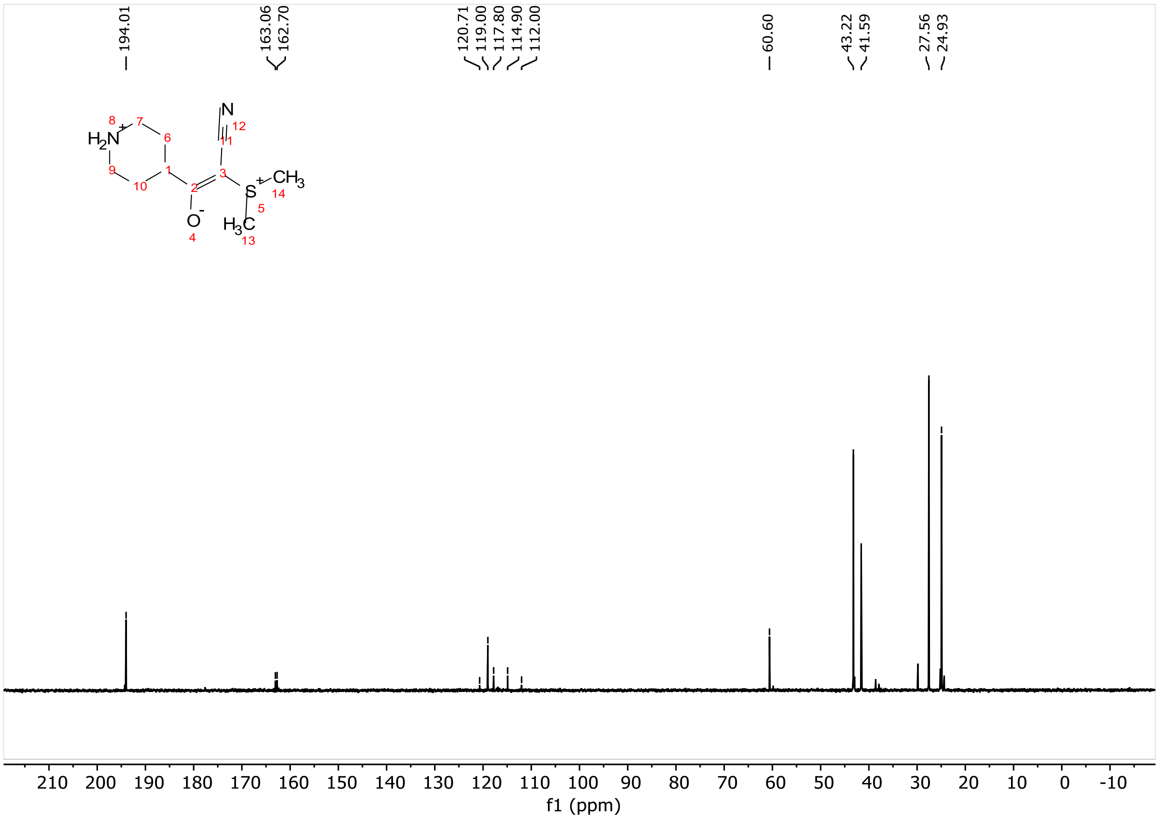

Figure S14. ^{13}C NMR spectrum (101 MHz, Deuterium Oxide) of the deprotected acrylamide sulfur ylide precursor **4**.

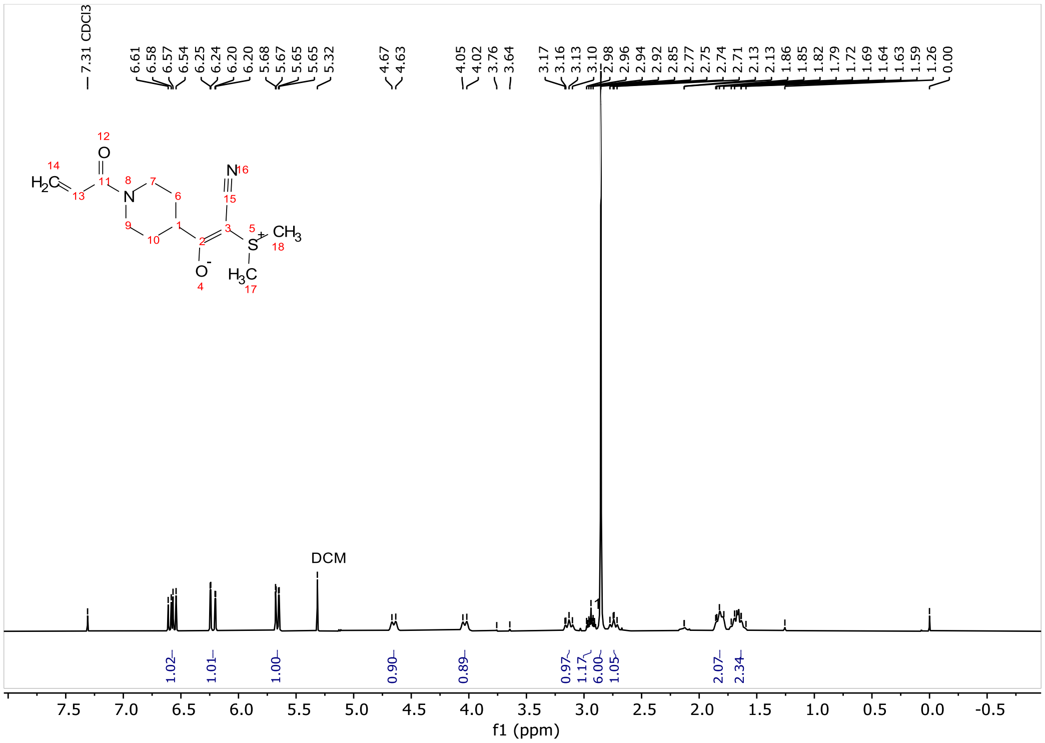

Figure S15. ^{1}H NMR spectrum (400 MHz, Chloroform-*d*) of the monomer **1**.

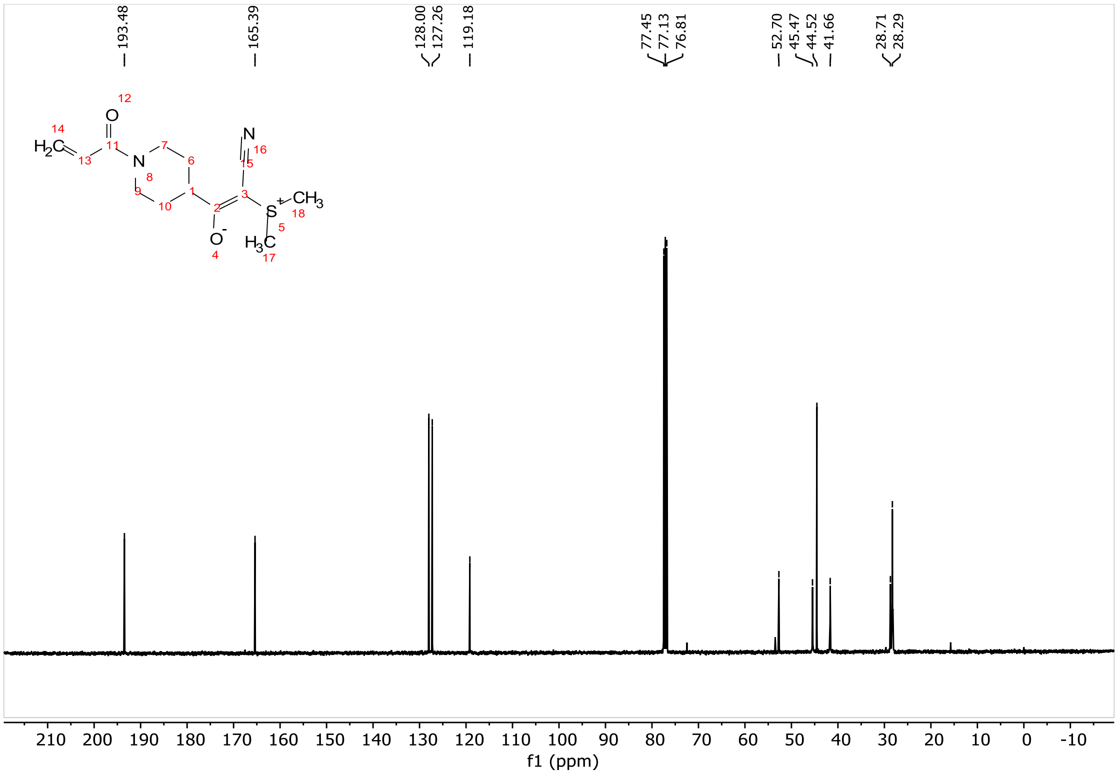

Figure S16. ^{13}C NMR spectrum (101 MHz, Chloroform-*d*) of the monomer **1**.

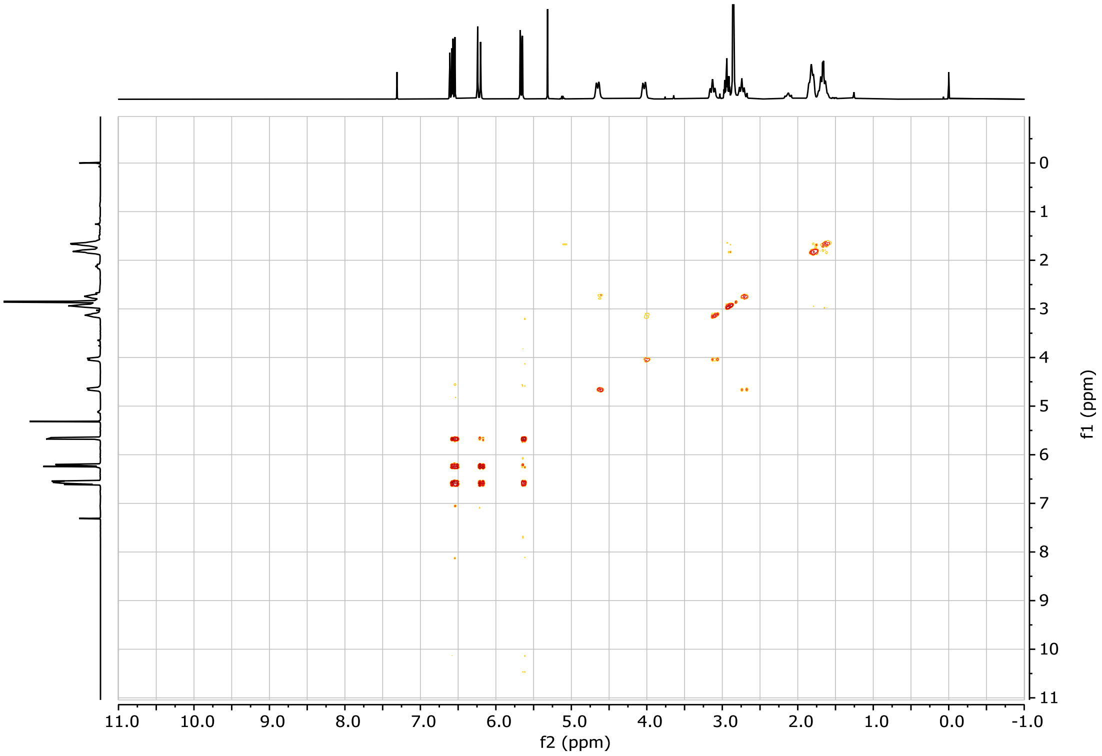

Figure S17. ^{1}H-^{1}H COSY NMR spectrum of the monomer **1**.

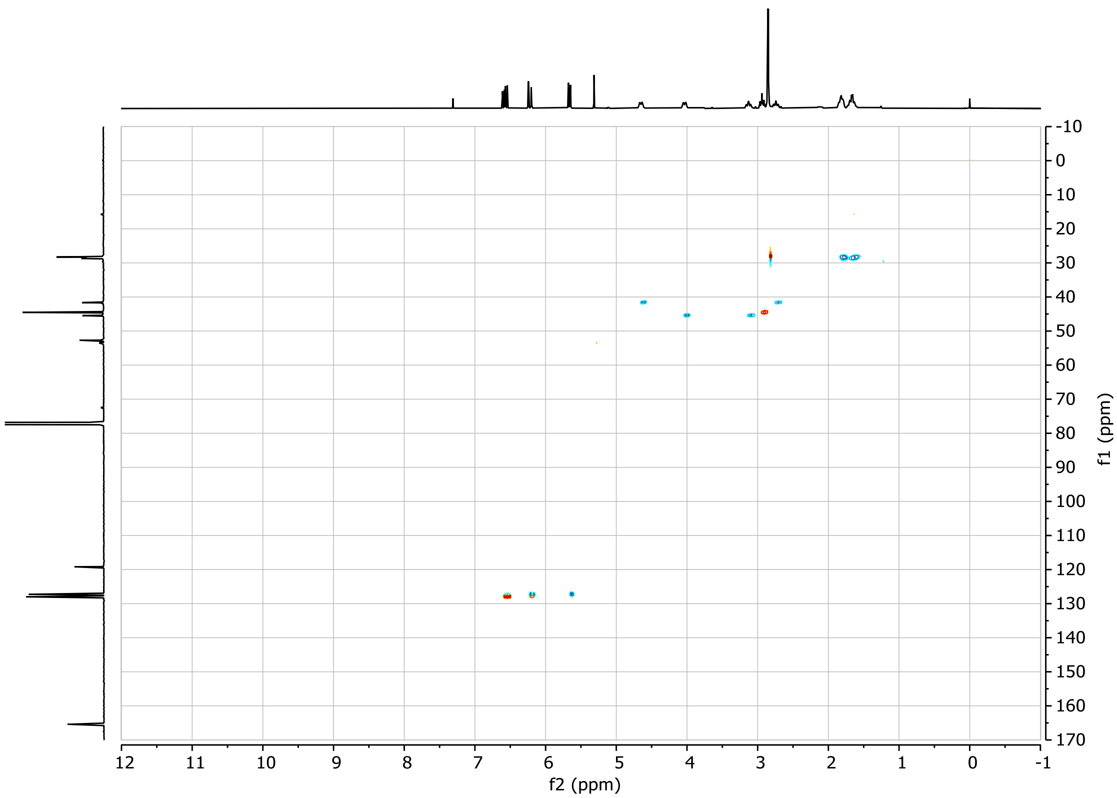

Figure S18. ^{1}H-^{13}C HSQC NMR spectrum of the monomer **1**.

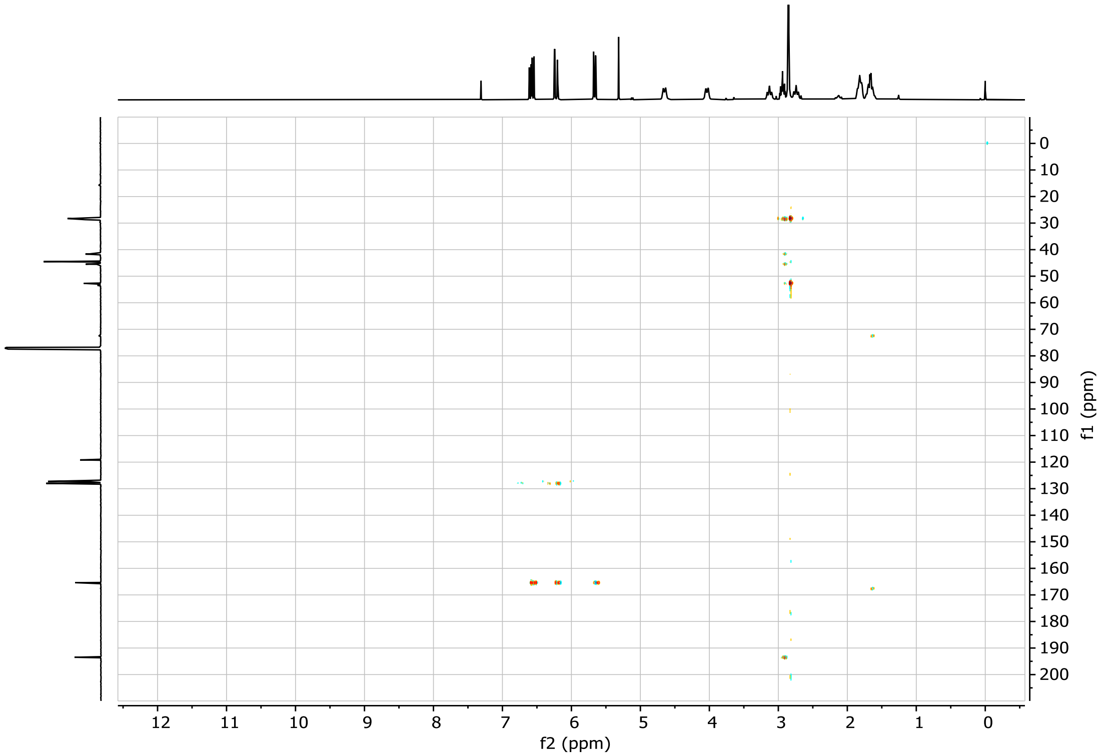

Figure S19. ^{1}H-^{13}C HMBC NMR spectrum of the monomer **1**.

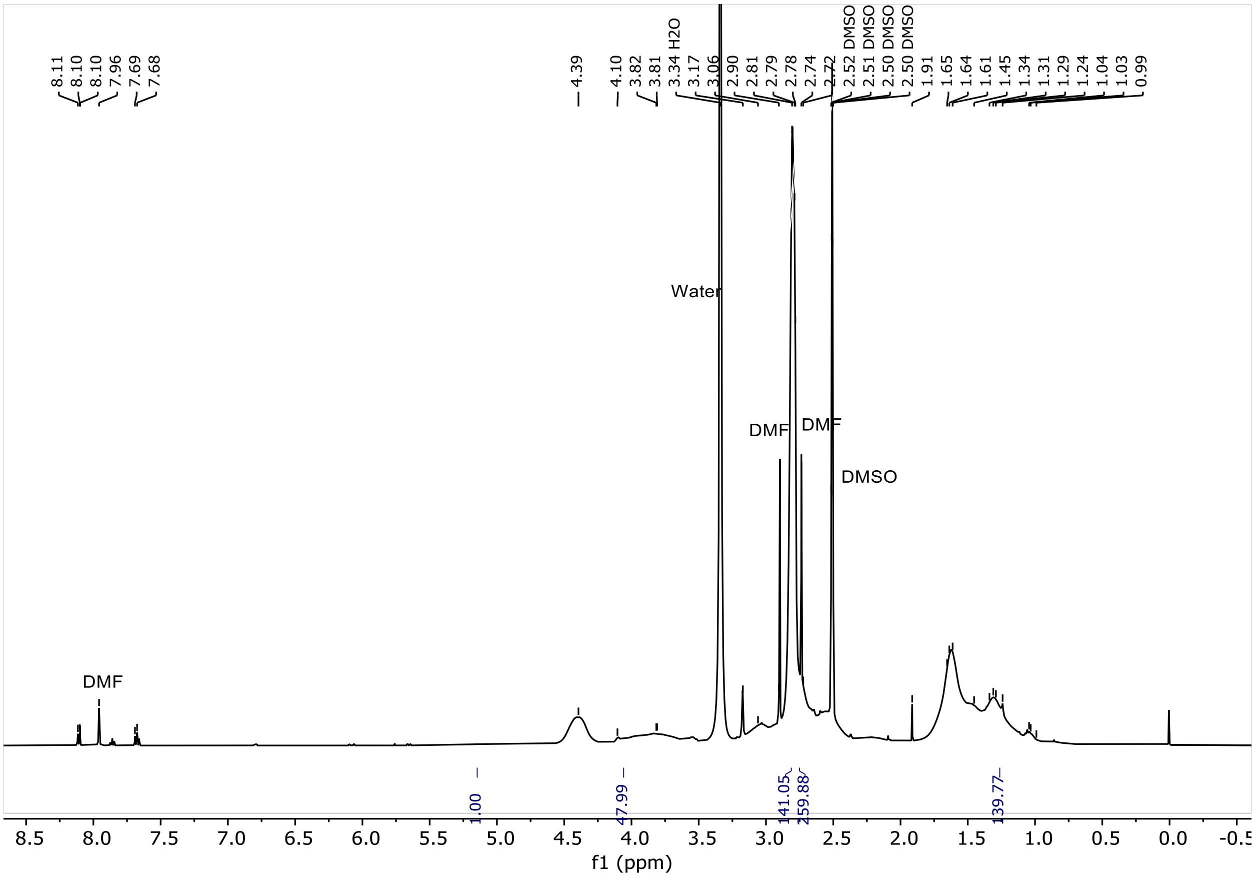

Figure S20. ^{1}H NMR spectrum (500 MHz, Dimethyl sulfoxide-d_6) of the Poly(SY-AAm).

9. References

(1) Berking, B. B.; Poulladofonou, G.; Karagrigoriou, D.; Wilson, D. A.; Neumann, K. Zwitterionic Polymeric Sulfur Ylides with Minimal Charge Separation Open a New Generation of Antifouling and Bactericidal Materials. *Angew. Chemie Int. Ed.* **2023**, *62* (41).

(2) Poulladofonou, G.; Neumann, K. Poly(Sulfur Ylides): A New Class of Zwitterionic Polymers with Distinct Thermal and Solution Behaviour. *Polym. Chem.* **2022**, *13* (30), 4416–4420.

(3) Van Oss, C. J.; Chaudhury, M. K.; Good, R. J. Interfacial Lifshitz-van Der Waals and Polar Interactions in Macroscopic Systems. *Chem. Rev.* **1988**, *88* (6), 927–941.

(4) Terzis, A.; Sauer, E.; Yang, G.; Groß, J.; Weigand, B. Characterisation of Acid–Base Surface Free Energy Components of Urea–Water Solutions. *Colloids Surfaces A Physicochem. Eng. Asp.* **2018**, *538*, 774–780.

(5) Rudawska, A.; Jacniacka, E. Evaluating Uncertainty of Surface Free Energy Measurement by the van Oss-Chaudhury-Good Method. *Int. J. Adhes. Adhes.* **2018**, *82*, 139–145.

(6) Invernizzi, M.; Parrinello, M.; Rethinking metadynamics: From bias potentials to probability distributions. *The Journal of Physical Chemistry Letters*, **2020**, 11, 7, 2731–2736

(7) Marzari, N.; Mostofi, A. A.; Yates, J. R.; Souza, I.; Vanderbilt, Maximally localized Wannier functions: Theory and applications, Rev. Mod. Phys., **2012**, 84, 1419.
